# The *Sinbad *retrotransposon from the genome of the human blood fluke, *Schistosoma mansoni*, and the distribution of related *Pao*-like elements

**DOI:** 10.1186/1471-2148-5-20

**Published:** 2005-02-23

**Authors:** Claudia S Copeland, Victoria H Mann, Maria E Morales, Bernd H Kalinna, Paul J Brindley

**Affiliations:** 1 Department of Tropical Medicine, Tulane University Health Sciences Center, New Orleans, USA; 2 Interdisciplinary Program in Molecular and Cellular Biology, Tulane University Health Sciences Center, New Orleans, USA; 3 Department of Molecular Parasitology, Institute for Biology, Humboldt University Berlin, Berlin, Germany

## Abstract

**Background:**

Of the major families of long terminal repeat (LTR) retrotransposons, the *Pao*/*BEL *family is probably the least well studied. It is becoming apparent that numerous LTR retrotransposons and other mobile genetic elements have colonized the genome of the human blood fluke, *Schistosoma mansoni*.

**Results:**

A proviral form of *Sinbad*, a new LTR retrotransposon, was identified in the genome of *S. mansoni*. Phylogenetic analysis indicated that *Sinbad *belongs to one of five discreet subfamilies of *Pao/BEL *like elements. BLAST searches of whole genomes and EST databases indicated that members of this clade occurred in species of the Insecta, Nematoda, Echinodermata and Chordata, as well as Platyhelminthes, but were absent from all plants, fungi and lower eukaryotes examined. Among the deuterostomes examined, only aquatic species harbored these types of elements. All four species of nematode examined were positive for *Sinbad *sequences, although among insect and vertebrate genomes, some were positive and some negative. The full length, consensus *Sinbad *retrotransposon was 6,287 bp long and was flanked at its 5'- and 3'-ends by identical LTRs of 386 bp. *Sinbad *displayed a triple Cys-His RNA binding motif characteristic of Gag of *Pao*/*BEL*-like elements, followed by the enzymatic domains of protease, reverse transcriptase (RT), RNAseH, and integrase, in that order. A phylogenetic tree of deduced RT sequences from 26 elements revealed that *Sinbad *was most closely related to an unnamed element from the zebrafish *Danio rerio *and to *Saci-1*, also from *S. mansoni*. It was also closely related to *Pao *from *Bombyx mori *and to *Ninja *of *Drosophila simulans*. *Sinbad *was only distantly related to the other schistosome LTR retrotransposons *Boudicca*, *Gulliver*, *Saci-2*, *Saci-3*, and *Fugitive*, which are *gypsy*-like. Southern hybridization and bioinformatics analyses indicated that there were about 50 copies of *Sinbad *in the *S. mansoni *genome. The presence of ESTs representing transcripts of *Sinbad *in numerous developmental stages of *S. mansoni *along with the identical 5'- and 3'-LTR sequences suggests that *Sinbad *is an active retrotransposon.

**Conclusion:**

*Sinbad *is a *Pao/BEL *type retrotransposon from the genome of *S. mansoni*. The *Pao/BEL *group appears to be comprised of at least five discrete subfamilies, which tend to cluster with host species phylogeny. *Pao/BEL *type elements appear to have colonized only the genomes of the Animalia. The distribution of these elements in the Ecdysozoa, Deuterostomia, and Lophotrochozoa is discontinuous, suggesting horizontal transmission and/or efficient elimination of *Pao*-like mobile genetic elements from some genomes.

## Background

*Schistosoma mansoni*, the African blood fluke and etiological agent of intestinal schistosomiasis, is endemic in numerous countries in Africa, the Middle East, the Caribbean and northeastern South America. The life cycle of *S. mansoni *involves parasitism of both humans and aquatic snails of the genus *Biomphalaria*. Cercariae, the infectious larvae, emerge from the snails into lakes and fresh water streams, where they initiate human infection by direct penetration of the skin. Within the infected person, the worms develop into male and female adults within the portal system blood vessels and mesenteric veins of the intestines. Eggs released from the female parasite into the blood traverse the intestinal wall and are passed out with the feces. Among the tropical diseases, schistosomiasis ranks second only to malaria in terms of morbidity and mortality [1] and has proved refractory to control by the more conventional public health approaches. No vaccine is yet available.

Mobile genetic elements (MGEs) represent a major force driving the evolution of eukaryotic genomes [2-4] and play an important role in the establishment of genome size [5]. One of the major categories of MGEs is the long terminal repeat (LTR) retrotransposable element, i.e. the LTR retrotransposons and the retroviruses [6]. These elements are of interest for their potential for horizontal transmission, as well as their ability to shed light on phylogenies of their host organisms when solely vertically transmitted. The genomes of schistosomes, blood flukes of the phylum Platyhelminthes, are estimated at ~270 megabase pairs (MB) per haploid genome [7], arrayed on seven pairs of autosomes and one pair of sex chromosomes [8,9]. Both the evolution and size of this genome may be highly influenced by mobile genetic elements. Indeed, more than half of the schistosome genome appears to be composed of, or derived from, repetitive sequences, to a large extent from retrotransposable elements [10,12]. Mobile genetic elements colonizing the genome of *S. mansoni *are of interest both for their potential in developing tools for schistosome transgenesis and for their influence on the evolution and structure of the schistosome genome [13,14]. Previously characterized schistosome MGEs include SINE-like retroposons [15,16], long terminal repeat (LTR) retrotransposons [12,17,18], non-LTR retrotransposons [10,11], and DNA transposons related to bacterial IS1016 insertion sequences [19]. *Boudicca*, the first LTR retrotransposon characterized from the genome of *S. mansoni *[20] belongs to the *gypsy *-like retrotransposons, one of three highly divergent groups of LTR retrotransposons: the *Gypsy/Ty3 *group, the *Copia/Ty1 *group and the *Pao/BEL *group [21]. Although active replication of schistosome retrotransposons has not been established, transcripts encoding reverse transcriptase (RT) and endonuclease are detectable [10,11,22], as is RT activity in parasite extracts [23], suggesting that at least some of these elements are actively mobile within the genome. Indeed, actively replicating MGEs have been described from other platyhelminths as RNA intermediates [24] and DNA transposons [25,26]. Furthermore, the schistosome retrotransposons characterized so far are highly represented within the genome with copy numbers of up to 10,000 [10,20].

It has been suggested that the *Pao*-like elements exhibit a host range limited to insects and nematodes [27]. More recently, however, *Pao*-like sequences have been reported from vertebrates including the teleost fishes *Takifugu rubripes *and *Danio rerio *[28]. Here we have characterized a new *Pao*-like element from the genome of *S. mansoni*, which we have named *Sinbad *after the mariner-explorer Sinbad from the classical Persian/Arabic tales of the "1001 Arabian Nights" (e.g., [29]). (Sinbad roved through near Eastern countries where schistosomiasis remains endemic even today [30].) Further, we investigated the phylogenetic distribution of *Pao*-like elements related to *Sinbad *and report that there is a discontinuous distribution of these elements throughout the Ecdysozoa, Deuterostomia, and Lophotrochozoa that suggests horizontal transmission and/or efficient elimination of *Pao*-like mobile genetic elements from some host genomes.

## Results

### A LTR retrotransposon in BAC 33-N-3

**Figure 1:**
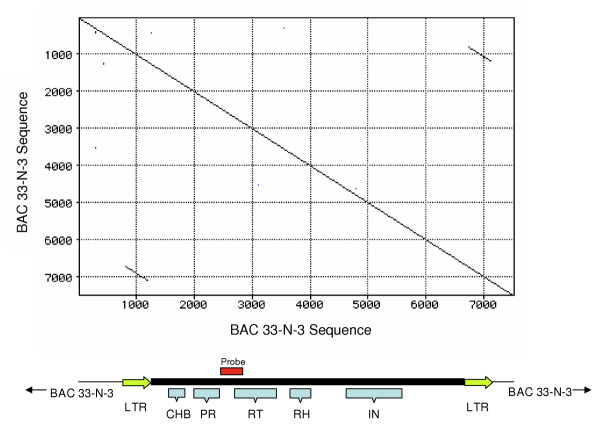
Pustell DNA matrix (DotPlot) of 7,531 bp of *Schistosoma mansoni *genomic DNA sequenced from BAC clone 33-N-3, revealing two identical 386 bp repeat sequences flanking 5,515 bp of unique sequence. A schematic representation of the 6,287 bp retrotransposon encoded by the sequence is shown below the matrix, with the positions of the LTRs and several domains (CHB, Cys-His Box; PR, protease; RT, reverse transcriptase; RH, RNaseH; IN, integrase) labeled. The position of the probe employed in library screening and Southern hybridization is indicated by a red box above this schematic representation. Both matrix and schematic are to scale.

BLAST analysis indicated the presence in BAC 30-H-16 of a reverse transcriptase (RT)-encoding sequence with identity to *Pao *and other *Pao*-like retrotransposons including *Ninja *and *MAX *(not shown). Using a probe based on an RT encoding segment of the end sequence of BAC 30-H-16, we identified 14 positive clones in the *S. mansoni *BAC library [31]. DotPlot analysis of a 7,531 bp portion of one of the positive BACs, 33-N-3, revealed the presence of two identical, direct repeat sequences of 386 bp separated by ~5.5 kb of intervening sequence, suggesting the presence of an LTR retrotransposon of 6,287 bp in length. This dot matrix is presented in Figure 1, with a map predicting the size and general domain structure of the new element provided below the matrix (both matrix and map share the same size scale). The direct repeats appeared to be LTRs, and included the promoter initiation motifs CAAT (positions 347–350) and TATA (positions 111–114 and 216–219), transcriptional signals for RNA polymerase II. The LTRs begin with TGT and end with TCA. These motifs (TGN/NCA), known as the direct inverted repeats (DIR), are common to LTRs of many retrotransposons and retroviruses [32]. BLAST searches of GenBank revealed that this retrotransposon closely resembled the elements *Pao *and *Ninja*, followed by other *Pao/BEL *type retrotransposons. We have termed this new retrotransposon *Sinbad*. The coding region between the two LTRs of *Sinbad *was disrupted by several stop and frameshift mutations (as has been seen in many other retrotransposons (e.g., see Ref. [32]), although the reverse transcriptase, retroviral protease, and *gag*-like domains of *Sinbad *were clearly evident. The sequence of the copy of *Sinbad *from BAC 33-N-3 has been assigned GenBank accession AY506538.

### *Pao*-like nucleoprotein, protease and reverse transcriptase

**Figure 2:**
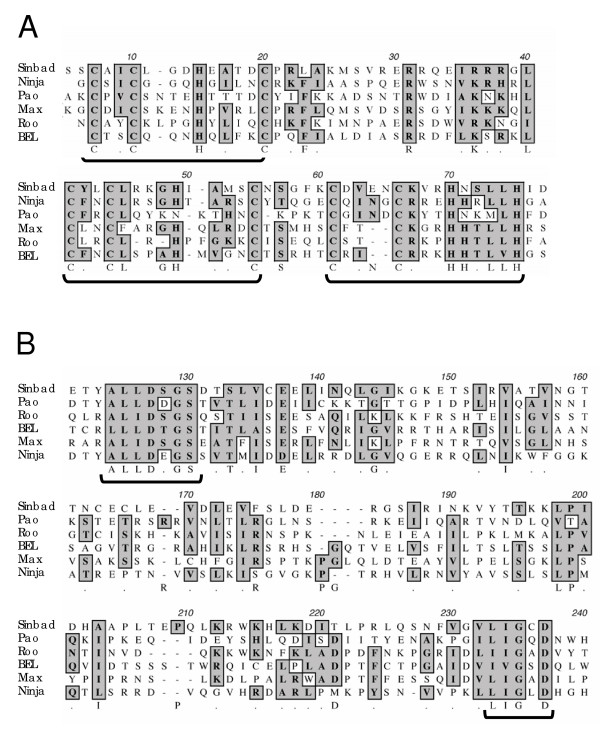
Multiple sequence alignments of key domains of the nucleocapsid protein and protease of the *Sinbad *retrotransposon and related elements. A. Amino acid alignment of the Cys-His box region of the nucleocapsid protein of *Sinbad *and five other *Pao*-like elements. *Sinbad *shares the triple Cys-His box motif of these elements (underlined). B. Amino acid alignment of the protease domain of *Sinbad *and five other *Pao*-like elements. *Sinbad *shares the **LLD**X**G **+ **LIG **protease motifs conserved in *Pao*-like elements (underlined). Identical and chemically similar residues are boxed and shaded.

Inspection of the region downstream of the 5'-LTR of *Sinbad *revealed the presence of an ORF encoding retroviral *gag *and *pol*-like proteins. A multiple sequence alignment of some of the key structural and enzymatic domains is presented in Figure 2, with the *Sinbad *sequence and orthologous regions from *Pao*, *Roo*, *BEL*, *MAX *and *Ninja*. The Cys-His box is a highly conserved cysteine and histidine based motif of the nucleocapsid protein (part of the *gag *polyprotein) of retroviruses and retroviral like elements [33]. Whereas many other retroviral and retrotransposon families exhibit Cys-His boxes based on a single or double motif of three cysteine and one histidine residues, *Pao*-like elements are characterized by a distinctive triple Cys-His box [21,27], with zinc finger motifs of **C**X2**C**X3-4**H**X4**C**, **C**X2**C**X2-4**H**X4-5**C**, and **C**X2-4**C**X3**H**X4**H**. *Sinbad *also exhibits the latter type, hallmark triple Cys-His box motif (Fig. 2, panel A), although neither *Sinbad *nor *Pao *shows a doublet HH in the middle of the third zinc finger motif, another characteristic of this group of retrotransposons [32]. Notably, *Tas*, a *Pao/BEL *like element from *Ascaris lumbricoides *does not share this characteristic triple Cys-His box [34], and though *Suzu *from *Takifugu rubripes *exhibits a triple Cys-His box, its third zinc finger motif exhibits the structure **C**X4**C**X6**HH**X3**C **[28]. As illustrated in Figure 2, panel B, *Sinbad *exhibited a protease domain motif **AL**L**D**S**GS**-X98-**LIG**C**D**, typical of the **LLD**X**G **and **LIG **protease motifs conserved in *Pao*-like retrotransposons [27]. The usual active site tripeptide motif in retroviral aspartic proteases is DTG, with a full conserved sequence of LLDTG, complemented by another site, a highly conserved G preceded by two hydrophobic residues, often I or L, which loops around to interact with the LLDTG [35]. Whereas the *Gypsy*-like and *Copia*-like elements exhibit DTG at the active site, *Sinbad *has DSG, as do two other *Pao*-like elements, *Roo *and *MAX*. Other *Pao*-like elements have even more divergent catalytic domains: DCG for *Kamikaze*, GDG for *Yamato*, and DNG for *Moose *[27]. Since only Thr and Ser include the alcohol groups required for catalysis [35], the non-DT/SG motifs, including the DDG and DEG of *Pao *and *Ninja *likely represent inactivating mutations in non-functional copies of the retrotransposons.

**Figure 3:**
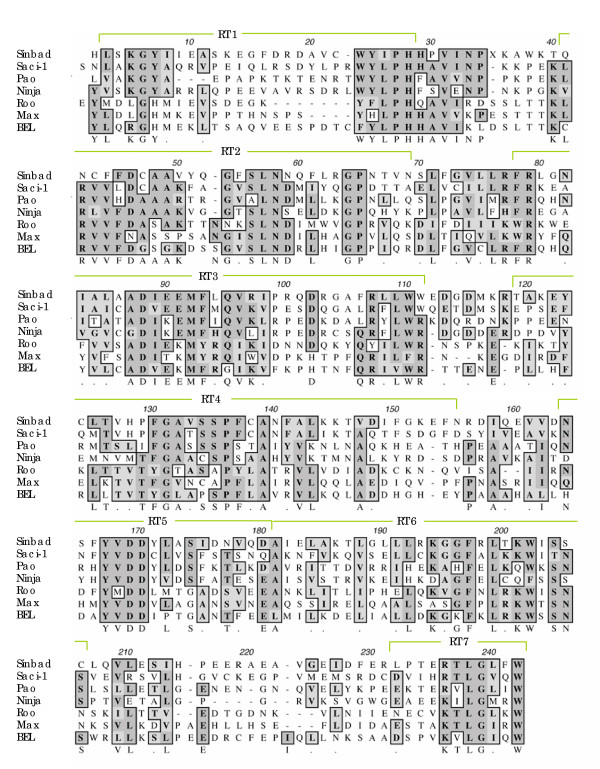
Multiple sequence alignment of deduced amino acid residues of the reverse transcriptase (RT) domain of *Sinbad *and six other *Pao*-like elements. Numbered blocks delineated by green brackets correspond to the seven conserved blocks of RT residues as described by Xiong et al. [21]. Identical and chemically similar residues are boxed and shaded.

Nucleotides 2761 to 3375 of the *Sinbad *sequence from BAC 33-N-3 encoded a RT domain, a conceptual translation of which was aligned with the RT domain from six other elements, *Pao*, *Ninja*, *Roo*, *BEL*, *Max*, and *Saci-1*. A frameshift apparent in the ORF was resolved by inserting a N at the frameshift site, position 2761. The seven blocks of conserved RT residues of *Pao*-like elements, as modified by Abe et al. [27] from the blocks described by Xiong et al. [21], are annotated in green in the alignment (Figure 3). The *Pao*-like retrotransposons presented in Figure 3 all exhibited the RT active site motif YV/MDD, in block 5, a motif conserved in the RT of many other retrotransposons, including the *gypsy *family [32].

### RNAse H and Integrase of *Sinbad*

**Figure 4:**
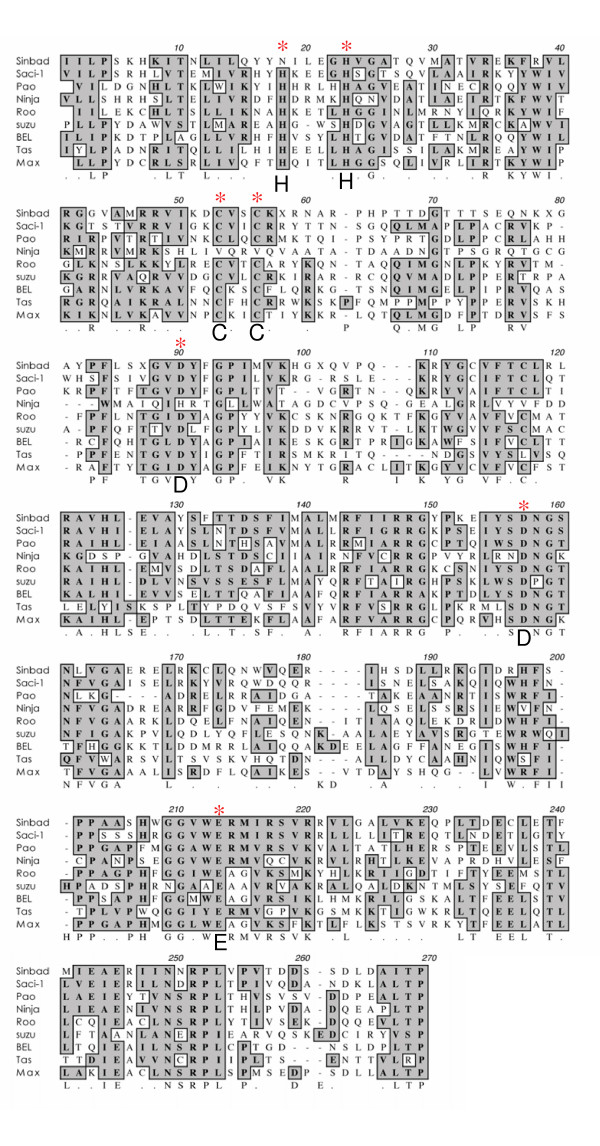
Multiple sequence alignment of deduced amino acid residues of the integrase (IN) domain of *Sinbad *from *Schistosoma mansoni *and eight other *Pao*-BEL family retrotransposons. The position of the active site residues are indicated with asterisks above and bold face letters (D, D or E) below, as are the key Cys (C) and His (H) residues of the zinc-finger motif. Identical and chemically similar residues are boxed and shaded.

An RNaseH domain spanning ~300 amino acid residues was located carboxyl to RT, in which the conserved active site motif DAS was apparent [see Additional file 1]. At its COOH-terminus, the *Sinbad *pol included an integrase (IN) domain of ~260 amino acids in length. Integrase mediates integration of a DNA copy of the viral genome into the host chromosome. Integrase is composed of three domains, the amino-terminal zinc binding domain, a central catalytic domain, and a carboxyl terminal domain that is a non-specific DNA binding domain [36]. A multiple sequence alignment of the IN zinc binding and central catalytic (DDE) domain of several informative *BEL/Pao*-like retrotransposons including *MAX*, *Saci-1*, *Pao*, *Ninja*, *Roo*, *Suzu*, *BEL*, *and Tas *as well as *Sinbad *is presented in Figure 4. All three domains were apparent in the *Sinbad *sequence. The NH_2_-terminal zinc-finger region of *Sinbad *included two conserved Cys residues and one His residue characteristic of other zinc finger motifs of IN (Figure 4). A second His expected here was replaced by Asn in this copy of Sinbad. The catalytic active site DDE motif of *Sinbad*'s integrase displayed the residue spacing of D(62)D(49)E. The IN of non-*Pao/BEL *retrotransposable elements, for example, *Copia*, exhibit a DD(35)E motif [36]. However, the IN of *BEL*/*Pao *like elements is unusual in that there is an expanded number of residues between the second D and E conserved residues, with DD(45)E for *Pao *and DD(53)E for *BEL*. *Sinbad *conformed to this *BEL*/*Pao*-like paradigm with a spacing of DD(49)E. *Saci-1*, also from *S. mansoni*, shows DD(49)E, although the IN domain of these two elements exhibited only 52% identity. The carboxy terminal domain of IN of *Sinbad *extended about 135 amino acids beyond the E residue of the catalytic domain [see Additional file 1].

As noted, the IN of *Sinbad *exhibited identity to *Saci-1 *from *S. mansoni*, and indeed these *Pao*-like retrotransposons from *S. mansoni *share substantial identity in deduced amino acid sequence and in structural organization [37]. This similarity extended to several other domains including the Triple Cys-His box region of Gag, 32% identical (23/71, Fig. 2A); PR,32% identical (36/111, Fig. 2B); and RT, 45% identical (106/236, Fig. 3). Whereas these levels of sequence identity confirmed a close relationship between *Sinbad *and *Saci-1*, they also demonstrated that *Sinbad *and *Saci-1 *are distinct retrotransposons. Finally, *Sinbad *did not appear to encode an envelope protein, the retroviral gene product necessary for extracellular existence and infection [38].

### *Sinbad*, a new *Pao*/*BEL *clade retrotransposon, is closely related to *Pao and Ninja*

**Figure 5:**
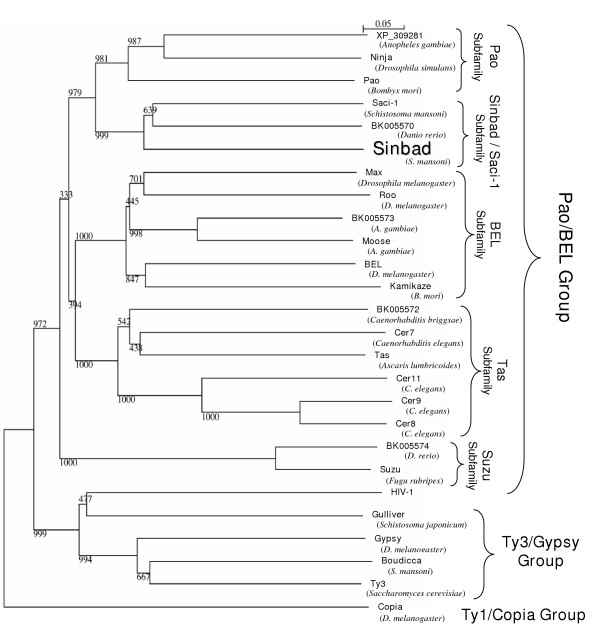
Phylogenetic tree based on Clustal X alignments of the reverse transcriptase domains of several *Pao*-like and non-*Pao*-like elements, drawn using the neighbor joining algorithm. The names of elements, followed by host species names, in parentheses, are provided. Size bar reflects phylogenetic divergence in genetic distance units. Bootstrap values were drawn from 1,000 trials.

The RT domain of *Sinbad *was aligned with that of 19 *Pao/BEL *retrotransposon family elements, and with RT from informative *Gypsy*-like elements, from HIV-1, and *Copia *using ClustalW. Bootstrapped trees were then assembled using the neighbor joining method and Njplot. *Copia *was employed as the outgroup to root the tree. The phylogenetic tree confirmed that *Sinbad *belonged to the *Pao*/*BEL *family of LTR retrotransposons (Figure 5), and revealed that its two closest relatives were the *Saci-1 *element from *S. mansoni *and an unnamed element from *D. rerio*, the zebrafish (BK005570). *Sinbad *also grouped closely with *Pao *and *Ninja*. *Sinbad *is clearly distinct from the *Gypsy*-like retrotransposons, including *Gulliver *of *Schistosoma japonicum *and *Boudicca *of *S*.*mansoni*. *Sinbad *is also clearly distinct from HIV-1, representative of vertebrate retroviruses, and from *Copia*, representative of the *Ty1/Copia *group of LTR retrotransposons. Among the 20 *BEL*/*Pao *family elements represented in the tree, it was possible to distinguish several subfamilies. First, the outlying subfamily was a clade including *Suzu *(from *T. rubripes*) and an unnamed element from zebrafish. These are the only two elements that we have observed in this subfamily, and both occur in fish genomes. The other two branches of these retrotransposons include *Pao*, on the one hand, and *BEL *on the other. Moreover, two subfamilies of elements were apparent within each of the *Pao *and *BEL *branches. For the *Pao *branch, one sub-family included *Pao *(from *B. mori*), *ninja *(from *D. simulans*) and an unnamed element from *Anopheles gambiae *(XP_3092181). These subfamily elements were all from insect genomes. The other subfamily included *Sinbad*, *Saci-1 *and the *D. rerio *element BK005570; this subfamily has elements from schistosomes (Phylum Platyhelminthes) and fish. On the *BEL *branch of the tree, the first subfamily includes elements solely from nematode genomes – *Tas *(*A. lumbricoides*), several *Cer *elements from *C. elegans*, and an unnamed element from *C. briggsae *(BK005572). The other branch included *BEL *itself (from *D. melanogaster*), *Kamikaze *(*B. mori*), *MAX *(*D. melanogaster*) and *Moose *from *A. gambiae*. Members of this fifth subfamily occurred only in insect genomes.

In addition, a phylogram of IN sequences was assembled from 14 *Pao*/*BEL *family retrotransposons. The tree displayed the same general topography of branches as the RT-based phylogram and supported our suggestion that there are (at least) five discrete sub-families of *BEL*-*Pao *family retrotransposons: *Tas*-like, *BEL*-like, *Pao*-like, *Sinbad/Saci-1*-like, and *Suzu*-like (not shown; tree available from corresponding author). In similar fashion to the RT based tree, *Sinbad *and *Saci-1 *were closely related to each other and to the IN from the unnamed *Pao*-element from zebrafish (BK005571).

### *Copies of Sinbad *interspersed throughout the schistosome genome

**Figure 6:**
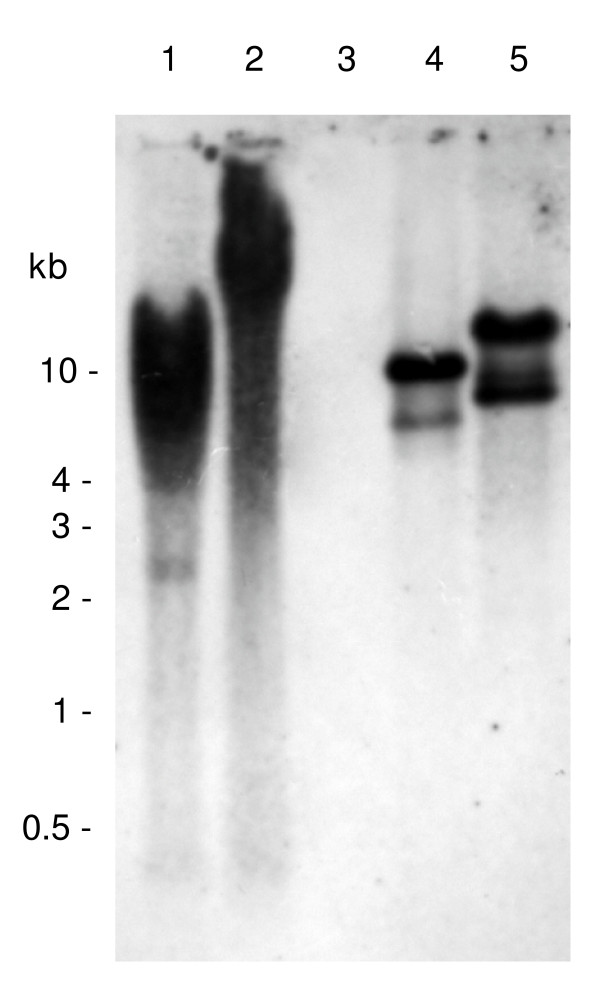
Southern hybridization of *Schistosoma mansoni *and *S. japonicum *genomic DNAs, and *S. mansoni *BAC clone 33-N-3 BAC DNA to a *Sinbad *retrotransposon-specific gene probe. Lane 1, *S. mansoni *DNA (30 μg) digested with *Hin*d III; lane 2, *S. mansoni *DNA (30 μg) digested with *Bam*H I; lane 3, *S. japonicum *DNA (20 μg) digested with *Hin*d III; lane 4: BAC 33-N-3 (0.8 μg) digested with *Hin*d III; and lane 5, BAC 33-N-3 (0.8 μg) digested with *Bam*H I. Molecular size standards in kilobase pairs (kb) are indicated at the left.

Southern hybridization analysis of *S. mansoni *gDNA, *S. japonicum *gDNA and BAC 33-N-3 confirmed the presence of *Sinbad *in the *S. mansoni *genome but indicated it was absent from the genome of the related schistosome, *S. japonicum *(Figure 6). *Bam*H I was expected to cut three times within *Sinbad*, whereas *Hin*d III, which cleaves the BAC 30-H-16 copy of *Sinbad*, was not predicted to cut within the sequence of the BAC 33-N-3 copy. The probe did not contain restriction sites for *Bam*H I or *Hin*d III. The hybridization signals from the two *S. mansoni *gDNA lanes (*Hin*d III or *Bam*H I digested) were strong and dispersed, with a band of ~2.6 kb in the *Hin*d III digest. The smeared pattern of hybridization indicated that a number of copies of *Sinbad *were interspersed throughout the genome of *S. mansoni *rather than being localized at a discrete locus. By contrast, the probe did not hybridize to the gDNA of *S. japonicum*. Additional blots with larger amounts (30 μg) of *S. japonicum *gDNA, digestion with *Bam*H I instead of *Hin*d III, and exposure of the film for longer periods failed to yield any signal from *S. japonicum *gDNA (not shown), indicating that *Sinbad *was absent from this schistosome species. Strong hybridization signals were evident in the positive control lanes of digests of BAC 33-N-3. Densitometric analysis of the hybridization signals indicated the presence of 50 to 60 copies of *Sinbad *per *S. mansoni *haploid genome, based on four separate estimates comparing the signal in each of the genomic DNA lanes to the signal in each of the 33-N-3 BAC lanes (comparison of lane 1 with lane 4, comparison of lane 2 with lane 5, comparison of lane 1 with lane 5, and comparison of lane 2 with lane 4). (These estimates assumed that BAC 33-N-3 included only one copy of *Sinbad*.)

Copy number was estimated by two additional methods. First, upon screening the 23,808 clones of the BAC library of Le Paslier et al. [31] that represents a ~8-fold coverage of the haploid *S. mansoni *genome, approximately 0.7% to 1.0% of the clones were positive, indicating a copy number for *Sinbad *of ~20 to 30 copies (not shown). Second, the bioinformatics approach of Copeland et al. [20] was used to compare these estimates with reference copy number estimates of other mobile genetic elements and genes reported previously. BLASTn searches were undertaken using the nucleotide sequences of these reference genes and the complete sequence of *Sinbad *(Table 1). Because the construction of the BAC library involved partial digestion of the genomic DNA with *Hin*d III [31], genes without *Hin*d III sites will be underrepresented in the BAC end sequences. Accordingly, since sequenced BAC ends from this library constitute a large proportion of the genomic *S. mansoni *sequences in the public domain, we used only genes containing *Hin*d III sites as reference sequences. As shown in Table 1, the number of hits for *Sinbad*, 38, was higher than the number of hits for the single-copy cathepsin D gene (0 hits) but lower than that for the multiple-copy 28S ribosomal RNA gene (157 hits) (~100 copies; Ref. [7]) and for three high copy number retrotransposons *Boudicca *(100 hits, 1,000–10,000 reported copies), *SR2 *(102 hits, 1,000–10,000 copies), and *SR1 *(104 hits, 200–2,000 reported copies). In overview, all three methods were in reasonably close agreement, and together they indicated that approximately 50 (range ~20–100) copies of *Sinbad *reside in the genome of *S. mansoni*. Based on copy numbers estimated for other schistosome retrotransposons (see [13]), we consider that *Sinbad *is not a high copy number element.

**Table 1:** Estimation of gene copy number of the Sinbad LTR retrotransposon in the genome of *Schistosoma mansoni*.

**Gene**	**GenBank Accession**	**Length **(bp)	**Number of hits (Expect 0.000001)**	**Reported copy number**	**Key references**
Cathepsin D, Intron 4	AY309267 (nt 3213–4849)	3926	0	1	66
***Sinbad***	**AY506538**	**6288**	**38**	**~50**	**This study**
28S rRNA	Z46503	1694	157	100	67
Boudicca	AY662653	5858	100	1,000–10,000	20
SR2	AF025672	3913	102	1,000–10,000	62
SR1	U66331	2337	104	200–2,000	61
Saci-2	BK004069	4946	107	85–850*	37
Saci-1	BK004068	5980	133	70–700*	37

*estimated solely by the gene index bioinformatics approach of DeMarco et al. [37], whereas the other copy numbers listed here were determined by hybridization and/or other analyses.

### *Sinbad*-like elements transcribed in developmental stages of *S. mansoni*

BLASTn analyses were undertaken using the full length of *Sinbad *as the query sequence and the GenBank EST database of non-human, non-mouse sequences. The database includes more than 130,000 EST sequences from six developmental stages of *S. mansoni *– egg, miracidium, cercaria, germball (= sporocyst), schistosomulum, and mixed sex adults [39,40]. Significant hits were found to ESTs from all of these six developmental stages. Of these, the hits with highest similarity to *Sinbad*, CD111741, CD060185, CD163413, CD062550, CD156994, and CD156946, exhibited contiguous ORFs spanning each EST without frameshifts or stop mutations. Positive ESTs spanning most or all of the LTR, *gag*, PR, RT, RH and/or IN regions were located in most of these six developmental stages, indicating that *Sinbad*-like elements are actively transcribed in all or most developmental stages of *S. mansoni*.

### Discontinuous distribution of *Sinbad*-like elements

In order to examine the phylogenetic distribution of *Sinbad*-like retrotransposons, we examined numerous complete and partial genomes, including prokaryotes, plants, fungi, animals, and lower eukaryotes [41]. The genomes were searched using tBLASTn with the amino acid sequence corresponding to the region of *Sinbad *spanning from the Cys-His box to the conserved protease catalytic domain (bp 1588–2236) [see Additional file 1] as the query. To minimize the likelihood of spurious positives, we lowered the E-value for significance from 10 to 0.001; this corresponded to a bit score of 40 or above. Although it is more stringent than that of the BLAST default, this cutoff point was employed because it is permissive enough to detect both *Sinbad*-like elements and members of the *Pao/BEL *family at large. No significant hits were found in any of the plant, fungal, or protist genomes examined, or in the 275 bacterial and 21 archaean genomes searched. All of the nematodes examined were positive. Of the insects, the other branch of the Ecdysozoa, *Drosophila melanogaster *and *Anopheles gambiae *contained *Sinbad*-like elements, whereas *Drosophila pseudoobscura *and *Apis mellifera *did not. Of the vertebrates, *Danio rerio *and *Takifugu rubripes *contained *Sinbad*-like sequences, whereas *Homo sapiens*, *Mus musculus*, *Rattus norvegicus, Canis familiaris, Sus scrofa, Gallus gallus*, and *Bos taurus *did not. Interestingly, although most higher chordates examined were free of *Sinbad*-like elements, the tunicates *Ciona intestinalis *and *Ciona savigny*, were positive for over 100 hits of sequences highly similar to the *Sinbad *search sequence (up to an E-value of 4e^-22^). In addition, the echinoderm *Strongylocentrotus purpuratus*, a non-chordate deuterostome, was positive for the *Sinbad *search sequence. These findings are summarized in a tree-of-life style illustration, based on the tree presented in Pennisi [42], and drawn in the style of the taxonomic relationship diagrams used at NCBI [43]. (This diagram is not a phylogram, and displays broad relationships among major taxa only; although relationships are in correct branching order, branch lengths are not to scale.) Genomes with regions of significant similarity to *Sinbad *are marked with a "+" symbol and those without are indicated with a "-" symbol. The results of a search of dbEST corroborated and expanded these findings, revealing nine non-*Schistosoma *organisms with *Sinbad *-like sequences: *C. intestinalis*, *Molgula tectiformis *(tunicate), *S. purpuratus*, *D. melanogaster*, *Bombyx mori*, *Salmo salar*, *Xenopus laevis*, and *Trichinella spiralis*. E-values and accession numbers for the top match for each organism are provided in Table 2.

**Table 2:** Organisms other than schistosomes with significant EST matches to *Sinbad*.

**Organism**	**Accession number**	**BLAST score (bits)**	**Expect value**
*Ciona intestinalis *(tunicate)	BW308116	89	9 e^-17^
*Molgula tectiformis *(tunicate)	AU283942	87	5 e^-16^
*Srongylocentrotus purpuratus *(purple sea urchin)	CD295138	83	7 e^-15^
*Drosophila melanogaster *(fruit fly)	BI583252	75	2 e^-12^
*Bombyx mori *(silk worm)	CK529741	59	1 e^-7^
*Salmo salar *(Atlantic salmon)	CB500934	58	2 e^-7^
*Xenopus laevis *(African clawed frog)	BJ073921	45	0.002
*Trichinella spiralis *(parasitic nematode)	BG520200	45	0.002

## Discussion

### *Sinbad *– a novel *Pao*/*BEL *family LTR retrotransposon from the genome of *S. mansoni*

Although several LTR retrotransposons have been characterized previously from the genome of *S. mansoni*, including *Boudicca*, *Saci-1*, *Saci-2*, *Saci-3 *and the *fugitive *[17,20,37], the *Sinbad *retrotransposon characterized here is a novel retrotransposon and it is discrete from these other elements. Sequence identity, structure, and phylogenetic relationships indicate that *Sinbad *is a member of the *Pao*/*BEL *family of retrotransposons. The hallmark structures included a triple Cys-His box zinc finger domain in the Gag polyprotein, protease with the active site tripeptide DSG, RT domain that included a YVDD active site motif, RNAseH with DAS at the active site, and an integrase domain with a DD(49)E spacing of the active site aspartic acid and glutamic acid residues. The YVDD motif of RT, a version of the F/YXDD consensus motif of *Gypsy*-like LTR retrotransposons, is shared by *Pao *and *BEL*. Bowen and McDonald [32] reported that the *Cer7-Cer12 *series of elements from *C. elegans *displayed YVDN at this site. Whether the Asn could replace Asp as the carboxy-residue of this conserved tetrapeptide with retention of enzyme activity remains to be determined by biochemical analysis, although mutation of either aspartate in YXDD of retroviral RT (HIV-1 or Moloney murine leukemia virus) inactivates the polymerase [see [44]].

The LTRs of *Sinbad *in BAC 33-N-3 are identical in sequence, and appeared to contain a putative promoter for initiation of transcription by RNA polymerase II. Along with conservation of most residues contributing to the active sites of the retrotransposon enzyme domains, these structural characteristics suggested that *Sinbad *is active or had been transpositionally active in the recent past. Several other features also indicated that *Sinbad *is transpositionally active. Numerous transcripts spanning enzymatic domains and LTRs of *Sinbad*, from at least six developmental stages of *S. mansoni*, have been sequenced [40], and of these, the ESTs most closely resembling *Sinbad *are composed entirely of contiguous open reading frames, suggesting non-mutated copies. On the other hand, potentially inactivating mutations, including stop codons and frameshifts, suggested that the BAC 33-N-3 copy of *Sinbad *was incapable of autonomous retrotransposition. If active copies are present, functional proteins coded by these copies could have been used in the recent past to mobilize the 33-N-3 *Sinbad *copy *in trans*, as recorded for other retrotransposons [45-47], explaining the presence of identical LTRs. Indeed, Frame et al. [28] noted that mutated copies framed by similar LTRs are common in *BEL *like elements in *C. elegans*, implying recent transposition.

**Figure 7:**
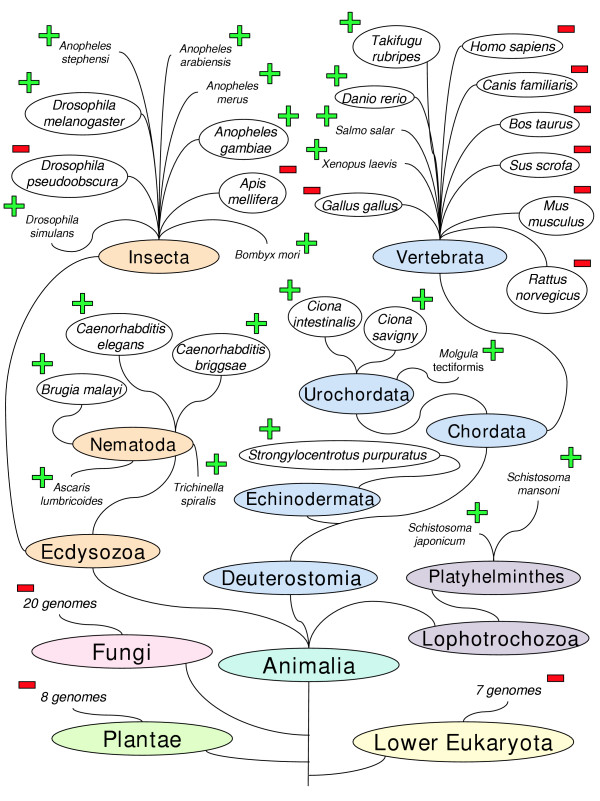
Phylogenetic illustration of species and higher taxa for which data are available concerning *Pao*-like elements. Species for which genomes have been sequenced and are available for whole genome BLAST searches in GenBank are enclosed in ovals. These genomes were tBLASTn searched using a deduced amino acid sequence from *Sinbad *(from the Cys-His Box through the protease domain) as the search sequence. Genomes with sequences significantly similar (E ≤ 0.001) to *Sinbad *are identified by a green "+" symbol, and those negative for *Sinbad*-like sequences with a red "-" symbol. Other species, with not yet fully sequenced genomes, shown to include *Pao*-like sequences (through EST searches or other means) are shown in smaller font and unenclosed, and are also marked with a green "+". This diagram is based on a tree of life style diagram in Pennisi [42] and reflects broad relationships between taxonomic groups only. It is not a phylogram – stem lengths do not represent phylogenetic distances.

The LTRs of *Sinbad*, at 386 bp in length, were substantially shorter than those of *Saci-1*, ~840 bp [37], but longer than those of *Gypsy*-like LTR retrotransposons from schistosomes, the *fugitive*, *Gulliver *and *Boudicca*. Whereas *Sinbad *and *Saci-1 *are clearly closely related, dissimilar LTRs and the low amino acid identity of the most highly conserved domains (35 to 52%) confirmed they are distinct retrotransposons. *Sinbad *can be added to the catalog of mobile genetic elements characterized from the schistosome genome, where retrotransposons appear to have proliferated and flourished and contributed significantly to its relatively large size (270 MB; ~14,000 protein-encoding genes) [13,20,40]. The colonization of the genome of *S. mansoni *by *Sinbad *and *Saci-1 *and that of *S. japonicum *by the related *Tiao *element [48] represents the first demonstration of infection of a Lophotrochozoan taxon by *Pao*/*BEL *family LTR retrotransposons. The presence of *Sinbad*, *Saci-1*, and *Tiao *in two species of *Schistosoma *suggests that an ancestral schistosome was already host to the ancestors of these elements. (Though *Tiao *is a *Pao/BEL *family retrotransposon, and is therefore predicted to be detected in low-stringency BLAST searches, as in Figure 7, the absence of a positive signal on the genomic Southern hybridization suggests that it is not particularly closely related to *Sinbad*.)

### A *Sinbad*/*Saci-1 *subfamily of *Pao-BEL *like LTR retrotransposons

Whereas the sequence and deduced structure of the three signature *Pao*-like elements, *Pao *from the silk moth *B. mori*, *Tas *from the human roundworm *Ascaris lumbricoides *and *BEL *from *D. melanogaster *have been known for about a decade, the *Pao*/*BEL *family is not as well understood or apparently as widespread as the other two major families of LTR retrotransposons, the *Copia*/*Ty1 *and the *Gypsy*/*Ty3 *families. However, at least three branches of the *Pao*/*BEL *family have become apparent – branches represented by *Pao*, *BEL*, and *Suzu *(from *T. rubripes*) [27,28,32,49-51]. Using the new sequence information from *Sinbad*, and some related elements, we have been able to investigate the intra-family relations of the *Pao*/*BEL *elements more thoroughly. Our findings, based on phylogeny of RT, and supplemented by phylogeny of IN, indicated the presence of at least five sub-families of *Pao*/*BEL *elements. The majority of the sub-families may have a restricted host range; the *Tas *subfamily occurred only in nematodes (these elements may be endogenous retroviruses because they appear to include *env *genes), the *BEL *subfamily only in insects, the *Pao *subfamily only in insects, and the *Suzu *subfamily only in fishes. By contrast, the *Sinbad/Saci-1 *subfamily is known from schistosomes and zebrafish.

### Phylogenetic range of *Sinbad*-like retrotransposons

The *Pao*/*BEL *retrotransposons are known only from animals, a less extensive distribution than those of the *Copia*/*Ty1 *or *Gypsy*/*Ty3 *groups that include elements known from fungi and/or plants as well as animals. The ostensible absence of these elements from prokaryotes, lower eukaryotes, fungi and plants suggests that ancestral *Pao*-like elements appeared after the differentiation of the Animalia. Though the number of sequenced entire genomes of animals is small, the distribution of *Pao*/*BEL *LTR retrotransposons within these few genomes displays a topography that we would not expect to be the result solely of vertical transmission alone (Fig. 7). *Sinbad*-like sequences were found in *D. melanogaster*, but not in *D. pseudoobscura*, nor in *A. mellifera*, even though close relatives are found in other insects such as *B. mori *and *A. gambiae*, and even in species as phylogenetically distant as *D. rerio *(a fish) and *S. mansoni *(a platyhelminth). Further, the distribution among chordates is enigmatic. Of the vertebrate whole genomes searched, only two, *T. rubripes *and *D. rerio*, were positive for *Sinbad *like elements. The human, mouse, rat, cow, chicken, pig and dog genomes were devoid of *Sinbad*-like matches. Since the genomes of lower chordates and a non-chordate deuterostome were positive for *Sinbad*-like sequences, progressive radiation would be expected to give rise to similar sequences in these vertebrates.

Feschotte [19] reported a similarly patchy distribution for the *Merlin *DNA transposons; *Merlin *like elements are abundant, for example, in anopheline mosquitoes but are absent from *D. melanogaster*, *D. pseudoobscura*, and *A. mellifera*. Also, they are present in some vertebrate genomes but not others. *Merlin*-like elements are also present in schistosome chromosomes. This type of distribution suggests that either the vertical lineage of the elements has been curtailed by the extinction of these elements from several genomes, or that horizontal transmission has taken place. Genomes need to restrain the uncontrolled proliferation of mobile genetic elements, especially retrotransposons, and indeed some eliminate mobile sequences more efficiently than others [5,52]. Goodwin and Poulter [53] have shown that *Ngaro *elements have been lost from certain genomes, as evidenced by the presence of small, corrupt fragments serving as fossil sequences. Similarly, especially in view of the low number of *Sinbad *copies, *Pao*-like elements may have followed a course of progressive radiation followed by elimination from the *Sinbad*-negative genomes. However, if this were the case with *Pao*-like elements, relic sequences could be expected in at least some of the *Sinbad*-negative genomes. Their absence from mammalian and avian genomes favors the alternative explanation, that the current range reflects horizontal transmission.

What might have been the origin of the *Pao*/*BEL *radiation within the Animalia? Felder et al. [34] suggested that a common ancestor of *Tas *and *Pao *may have undergone a horizontal transmission event between the Insecta and Nematoda, followed by the eventual differentiation of these elements, including the gain or loss of *env*. Of the sub-families of *Pao*/*BEL *elements apparent in the RT-based phylogram (Figure 5), the *Tas *subfamily includes retrotransposons with an envelope encoding gene (specifically *Tas *from *A. lumbricoides *and Cer7 from *C. elegans*). The acquisition of an envelope protein by an ancestral *Tas *or *Tas*-like element would have enabled its extracellular existence and facilitated its horizontal transmission and infection of other hosts [38].

Interestingly, the deuterostomes bearing *Sinbad*-like sequences included a sea urchin, tunicates, pufferfish, zebrafish, the Atlantic salmon, and the African clawed frog *X. laevis *(Figure 7). These are aquatic species and, moreover, all are known from coastal or brackish waters at the interface of freshwater and marine systems. The secondary hosts of *S. mansoni*, snails of the pulmonate genus *Biomphalaria*, are also aquatic, as are the larval (miracidium and cercaria) stages of *S. mansoni *which enter and exit the snail. It will be of interest to determine whether or not *Pao*-like elements are present in this snail host, from which numerous RT-encoding sequences already have been reported [54]. Also of potential relevance is that the genomes of both *X. laevis *and *S. mansoni *contain *Pao*-like elements and that *X. laevis *is the secondary host of the trematode parasite *Tylodelphys xenopi *[55], a fluke closely related to the human schistosomes. Both *T. xenopi *and another human schistosome, *Schistosoma haematobium*, use snails of the genus *Bulinus *as intermediate hosts. An aquatic lifestyle is an obvious relationship that links all of the deuterostome hosts of *Sinbad*-like elements. This aquatic, in comparison to a terrestrial, existence may have facilitated transmission of infectious particles of the *Tas*-like ancestors of *Pao*, *Tas*, *BEL*, *Suzu*, *Sinbad*, and relatives. Alternatively, schistosomes may have acquired a *Tas*- element directly from *Ascaris lumbricoides*, an exceedingly common human parasite and the host of *Tas*. *A. lumbricoides *occurs in the intestines of infected people, as do schistosome eggs, so direct transmission of a mobile genetic element from roundworm to schistosome could have been facilitated by their physical proximity within the human intestines.

## Conclusion

A *Pao*/*BEL *like LTR retrotransposon named *Sinbad *is interspersed within the genome of the blood fluke, *S. mansoni*. About 50 copies of this element appear to reside in the *S. mansoni *genome. Analyses of the phylogenetic distribution of *Pao*/*BEL*-like retrotransposons indicated that *Pao*/*BEL*-like elements are present only within phyla of the Animalia, and not in prokaryotes, fungi or plants. Further, the analyses indicated that there are at least five discrete sub-families of the *Pao*/*BEL *clade of LTR retrotransposons, and that the distribution of these retrotransposons among the Ecdysozoa, Lophotrochozoa and deuterosomes has been influenced by horizontal as well as vertical transmission.

## Methods

### Screening the bacterial artificial chromosome library

Le Paslier et al. [31] described the construction and characterization of a bacterial artificial chromosome (BAC) library of the *Schistosoma mansoni *genome. The library, constructed in the plasmid vector pBeloBac11 with genomic DNA (gDNA) from cercariae of a Puerto Rican strain of *S. mansoni *partially digested with *Hin*d III, consists of 23,808 clones, about 21,000 of which are estimated to contain inserts ranging from 120 to 170 kb, providing ~8-fold coverage of the schistosome genome. Numerous BAC end sequences determined from randomly selected clones from this library are in the public domain. Inspection of the end sequence of BAC clone number 30-H-16 indicated identity with *Pao*-like LTR retrotransposons (not shown). Because the retrotransposon sequence was located at the end of the BAC, the clone was unlikely to contain the entire *Pao*-like element. Given that retrotransposons can be expected to be present in multiple copies in the host genome, we screened the library with a probe based on the end of BAC 30-H-16 in order to locate an entire copy of the retrotransposon. The gene probe was obtained by PCR amplification of a fragment of BAC 30-H-16 using the primers 5'-CGCGGATCCAAGAGAAAAACCTTGATAGAC and 5'-CCGGAATTCCTGTCGAAGATAAAAGAGC, was cloned into pBluescript and its identity confirmed by sequencing (Accession AY871176). This probe spanned residues 2457 to 2823 of the BAC 33-N-3 copy of the new retrotransposon (see below). The cloned insert was labeled with digoxygenin (DIG) and employed to screen the BAC library, as described [20], represented as high-density clone arrays on nylon membranes. Positive clones were cultured as described [31] and the presence of sequences with identity to the novel retrotransposon in the positive clones was confirmed by PCR (primers as above) or by colony hybridizations [56] to the DIG labeled probe. One positive clone, BAC 33-N-3, was investigated further by sequence analysis. BAC plasmid DNA was isolated from bacterial cultures using the PhasePrep BAC DNA purification system (Sigma). Analysis of the insert of 33-N-3 was accomplished after subcloning *Bam*H1 fragments of the BAC into pNEB 193 (New England Biolabs, MA), sequencing the inserts of the sub-clones, and also by direct sequencing of BAC 33-N-3. Automated nucleotide sequencing, using ABI BigDye Terminator chemistry (ABI, Foster City, CA) and an ABI Prism 3100 sequencer, was undertaken using primers specific for the probe and subsequently with gene specific primers at Tulane University and at Davis Sequencing (Davis, CA).

### Sequence analysis and alignments

Contigs of the sequences were assembled using SeqMan (DNAstar, Inc., Madison, WI). Repeat sequences were identified with a Pustell style dot matrix [57] using the DotPlot3 program (Ramin Nakisa, Imperial College, London, UK) [see [58]] and the Pustell DNA Matrix function in MacVector (Accelrys). Amino acid alignments were accomplished with MacVector and ClustalW [59] using sequences from GenBank or using conceptual translations of nucleic acid sequences. Open reading frames were located and conceptually translated using MacVector. Sequences of the following retrotransposons were used in the multiple sequence alignments based on *gag*, protease, and reverse transcriptase: *Ninja*, T31674; *Pao*, S33901; *MAX*, CAD32253; *Roo*, AAN87269; *BEL*, AAB03640; and *Saci-1*, BK004068. Sequences of the following retrotransposons were used in the multiple sequence alignment based on Integrase: *Saci-1*, DAA04498;*Pao*, S33901; *Ninja*, T31674; *Roo*, AAN87269; *Suzu*, AF537216, *BEL*, AAB03640, *Tas*, Z29712, and *MAX*, CAD32253.

### Parasite DNAs, Southern hybridization, densitometric estimation of copy number

Genomic DNAs of cercariae of a Puerto Rican strain of *S. mansoni *and of adults of a Chinese (Anhui Province) strain of *S. japonicum *were extracted using the AquaPure Genomic DNA Purification system (Bio-Rad, Hercules, CA). *S. mansoni *gDNA (30 μg/lane) and 33-N-3 BAC DNA (800 ng) were digested with *Hin*d III and *Bam*H I restriction enzymes, and *S. japonicum *gDNA (20 μg/lane) was digested with *Hin*d III. Digested gDNA and BAC DNA were size fractionated by electrophoresis through a 0.8% agarose gel, transferred to a nylon membrane (Zeta-Probe GT, Bio-Rad) by capillary action [60], and UV-light cross-linked to the membrane. Southern hybridization analysis to the DIG-labeled probe (above) was performed as described [20]. Chemiluminescent signals were detected using X-ray film (Fuji). Densitometric analysis of Southern hybridization signals was accomplished using the Versa-Doc gel documentation system (Bio-Rad) and Quantity-One software (Bio-Rad). Densitometry values for signals evident in the gDNA and BAC DNA lanes were used to estimate the copy number for the new retrotransposon, *Sinbad*, according to the formula [(*A*/*B*) × *C*]/*E *= *F*. This formula was derived from two equations: (*A*/*B*) × *C *= *D *and *D*/*E *= *F*, where *A *was the number of copies of *Sinbad *in the BAC 33-N-3 lane, *B *was the density volume of the 33-N-3 lane in units of optical density per mm^2^, *C *was the density volume of the *S. mansoni *genomic DNA lanes in units of optical density per mm^2^, *D *was the total number of copies of *Sinbad *per genomic DNA lane, *E *is the number of haploid genomes in the gDNA lane, and *F *represented the copy number of *Sinbad *per haploid *S. mansoni *genome. The insert of 33-N-3 was estimated to be 145 kb in length and assumed to contain only a single copy of the retrotransposon.

### Other copy number estimations

In addition to the densitometry-based estimate, estimates of the copy number of the *Sinbad *retrotransposon also were obtained by a comparative bioinformatics approach [20] wherein BLAST analysis of the bacterial artificial chromosome (BAC) -end database of *S. mansoni *genomic sequences targeted more well-characterized retrotransposable elements from *S. mansoni *for which copy numbers had been reported. These included the *Boudicca *LTR retrotransposon [20] and the non-LTR retrotransposons *SR1 *and *SR2 *[61,62]. The NCBI database was searched by BLAST using the sequences of these mobile genetic elements and some other genes of *S. mansoni*, all of which included at least one *Hin*d III site. Specifically, the Advanced BLAST function was used, set to search only the *S. mansoni *sequences in the GSS database (Limit by Entrez Query: <Schistosoma mansoni[organism]>), and with the E value at 0.000001. The E value (Expect value) reflects the probability of obtaining a match purely by chance. Scores at or below this stringent cutoff E value of 10^-6 ^were counted as positive. This exceptionally stringent cutoff value was used to minimize the chance of counting other *Pao*-like elements in the total copy number of *Sinbad*. Since the formula for E is based not only on the bit scores of the local alignment of each pair of sequences, but also on the lengths of the subject and query [see [63]], no additional correction was made for the length of the query sequence.

### Phylogenetic analysis of *Pao*-like elements

Sequences for phylogenetic analysis comparing the RT region of several different retrotransposons were prepared by trimming sequences from the large single polyprotein of each retrotransposon to just the conserved domains of RT (see [21,27]). Pol sequences presented in Xiong et al. [21] and Abe et al. [27] were trimmed exactly to the stretch of sequence shown by these authors to represent the RT domain. Other elements were aligned with these sequences and likewise trimmed to obtain just the RT domain. For some elements, nucleotide sequences were analyzed for open reading frames and translated before being trimmed to include just the 7 conserved blocks of the RT domain. Alignments were accomplished using Clustal X [64], after which bootstrapped trees (1,000 repetitions) were prepared using the neighbor joining method [65] and drawn with Njplot. The accession numbers for sequences included in the phylogenetic analysis are as follows: *Ty3*, S53577; *Tas*: Z29712; *Suzu*, AF537216; *Sinbad*, AY506538 (an N was inserted at position 2761 to a resolve a frameshift and generate a single ORF) *Saci-1*, DAA04498; *Roo*, AAN87269; *Ninja*, T31674; *Moose*, AF060859; *Max*, CAD32253; *Kamikaze*, AB042120; HIV-1, P04585; *Gypsy*, GNFFG1; *Gulliver*, AF243513; *Copia*, OFFCP; *BEL*, AAB03640; Cer7, AAB63932, Cer8, CAB04994, Cer9, CAB1647, and Cer11, AAA82437, two uncharacterized *Anopheles gambiae *retrotransposons, XP_309281 and XM_308737, an uncharacterized *Caenorhabditis briggsae *retrotransposon, AC084491, and two uncharacterized *Danio rerio *retrotransposons, BX537152 and BX005079 [see Additional file 2]. Two additional sequences were either not in the database or were composites made to reconstruct sequences more closely resembling non-mutated forms of the retrotransposons. The sequence representing *Pao *was a reconstruction prepared by Abe et al. [27], from accession numbers S33901, AB042118, and AB042119; the sequence representing *Boudicca *was a composite of translated cDNA sequences introduced in Copeland et al. [22], AY308018, AY308019, AY308021 and AY308022 [see Additional file 2].

### Screening entire or partial genomes for *Sinbad*

A panel of fully or partially sequenced entire genomes was searched by BLAST for elements exhibiting sequence similarity to *Sinbad*. The deduced amino acid sequence encoding the region from the Cys-His Box through to the protease domain (encoded by nucleotides 106 to 753 of *Sinbad *[Y506538]) was employed as the query to search each genome individually using tBLASTn. The genomes searched in this way were as follows: *Homo sapiens, Mus musculus, Rattus norvegicus, Takifugu rubripes, Danio rerio, Bos taurus, Gallus gallus, Sus scrofa, Canis familiaris, Anopheles gambiae, Apis mellifera, Drosophila melanogaster, Drosophila pseudoobscura, Brugia malayi, Caenorhabditis elegans, Caenorhabditis briggsae, Strongylocentrotus purpuratus, Ciona intestinalis, Ciona savigny, Giardia lamblia, Plasmodium falciparum, Plasmodium yoelii, Plasmodium berghei, Cryptosporidium parvum, Eimeria tenella, Theileria annulata, Toxoplasma gondii, Dictyostelium discoideum, Entamoeba histolytica, Leishmania major, Trypanosoma brucei, Trypanosoma cruzi, Arabidopsis thaliana, Avena sativa, Glycine max, Hordeum vulgare, Oryza sativa, Triticum aestivum, Zea mays, Lycopersicon esculentum, Schizosaccharomyces pombe, Saccharomyces cerevisiae, Saccharomyces paradoxus, Saccharomyces mikatae, Saccharomyces bayanus, Saccharomyces castelli, Saccharomyces kluyveri, Saccharomyces kudriavzevii, Neurospora crassa, Magnaporthe grisea, Aspergillus nidulans, Aspergillus fumigatus, Aspergillus terreus, Candida albicans, Coccidioides posadasii, Gibberella zeae, Coprinopsis cinerea, Cryptococcus neoformans, Ustilago maydis *and *Encephalitozoan cuniculi*. In addition, 275 eubacterial and 21 Archaean genomes were searched [see Additional file 3]. Genomes with matches with E values less than 0.001 (corresponding approximately to bit scores greater than 40) were considered positive for *Sinbad*-like elements.

### GenBank accession numbers

Sequences of the *Sinbad *LTR retrotransposon have been assigned accession numbers AY506537, AY506538, AY645721, AAT66412, and AY871176. Other sequences introduced here been assigned GenBank Third Party Annotation accession numbers; BK005570 (*Danio rerio*), BK005571 (*D. rerio*), BK005572 (*Caenorhabditis briggsae*), BK005573 (*Anopheles gambiae*), BK005574 (*D. rerio*).

## Abbreviations

MGE, mobile genetic element; ORF, open reading frame; EST, expressed sequence tag; gDNA, genomic DNA; LTR, long terminal repeat; RT, reverse transcriptase; PR, protease; IN, Integrase; CHB, Cys-His box; BAC, bacterial artificial chromosome; MB, megabase pairs

## Authors' contributions

CSC carried out the sequence analyses, sequence alignments, phylogenetic studies, other bioinformatics analyses, and Southern hybridizations, participated in cloning, sequencing and design of the study, and, together with PJB, drafted the manuscript. VHM and MEM participated in cloning and sequencing. BHK contributed to the design of experiments and analyses. PJB participated in the design and coordination of the study, and drafting the manuscript. All authors read and approved the final version of the manuscript.

## Supplementary Material

Additional File 1 "Annotated *Sinbad *sequence". Nucleotide and deduced amino acid sequence of the entire *Sinbad *retrotransposon in BAC clone 33-N-3. Hallmark features of the retrotransposon are identified in colored highlights as described in the key at the bottom of the figure. Click here for file

Additional File 2 "RT domain sequences of new and consensus elements used in the phylogenetic analysis". Deduced amino acid sequences of the RT domains used in the phylogenetic analysis from newly characterized elements, uncharacterized elements found within genome survey sequences, and elements for which consensus sequences were used. Accession numbers for the source sequences of each element are listed, as well as references where applicable. Click here for file

Additional File 3 "Prokaryotic genomes negative for *Sinbad *like elements" Table of prokaryotic genomes indicated by whole genome analysis to be devoid of *Sinbad *like elements. Click here for file
