# Supplementary material for: The Sinbad retrotransposon from the genome of the human blood fluke, Schistosoma mansoni, and the distribution of related Pao-like elements
Source: BMC Evol Biol. 2005 Feb 23;5:20. doi: 10.1186/1471-2148-5-20 (PMC554778; doi:10.1186/1471-2148-5-20)
Supplement: Additional File 1 — "Annotated Sinbad sequence". Nucleotide and deduced amino acid sequence of the entire Sinbad retrotransposon in BAC clone 33-N-3. Hallmark features of the retrotransposon are identified in colored highlights as described in the key at the bottom of the figure. [file 1471-2148-5-20-S1.pdf]

Sequence Range: 1 to 6287

```
TGTTACGGGAGAACTAAAGACATTTGGCTGATTAAAACCTTTTTACTGTACATCAAATTTACTGTGTTTTTGGATGTGTTTGTTTTACTTAACCTTTGGA  100
CTTGTTTGTTTATATCATTCCTTTCGGACATTTTGGTTTCTTTTGTCATTGGTTTGGTATTTGGGAGTTTTTTGCTCCGGGAACCTACAAAATTGAAGGT  200
TTGTTTTGTAATTGTTATAATTATTGTTGTTAGAACGACTTTGGTCGATTATTTGTGAATTGCGAATAAACTTGGTATCTTATTTGGAGTTTTGGTTCTC  300
TTGCCTACTTGGGTTCGTAATTTGCGAATATCGACGGCCAGTACTTCAATAGTTGGAACGGGCAAAACTCATAATTGGACGTAACATAAAGTTCTTCAAT  400
                                                                                                      H  K  V  L  Q>

TATTGGTTCTGTGTTCACACTGGATTAATCTGGACAGTAATTTGGGTCCAAATTCTGGTTCTACTGAACGAAGGTGTACTATGGAAACTCGAAGCAAAAG  500
L  L  V  L  C  S  H  W  I  N  L  D  S  N  L  G  P  N  S  G  S  T  E  R  R  C  T  M  E  T  R  S  K  R>

ACAGAAGGAAAACGACGAATTGGACGGTAAAATTTCCAGAGCTGTTTGTGGTATGTATGACGTTAACGATGATAAATGTGATGATATTAGTCTTAATAGT  600
 Q  K  E  N  D  E  L  D  G  K  I  S  R  A  V  C  G  M  Y  D  V  N  D  D  K  C  D  D  I  S  L  N  S>

ATGTCCTCTAGCCAGCTAAGTACTTCTAGATTAAAGCTAGACAAAGCATTACTGAGGAAAAAGAATTTAGAAAAGAGACTTGAGTGGAACGGCAACTAAG  700
 M  S  S  S  Q  L  S  T  S  R  L  K  L  D  K  A  L  L  R  K  K  N  L  E  K  R  L  E  W  N  G  N  *>
                                                                                K  R  D  L  S  G  T  A  T  K>
ATGTTAGATCTTCAAGAAGAGGTTGATATGGCCGAACTAGAATGCAACGTCATCAAGGACGATGAACGGTTGCCCGTAGATAAATGTGAAATAAATGCTA  800
 M  L  D  L  Q  E  E  V  D  M  A  E  L  E  C  N  V  I  K  D  D  E  R  L  P  V  D  K  C  E  I  N  A>

AAGTGGAAAATTATTTGAATGGTGAAACGGGGGGTAATAATCACTCAGGAGAGGTATGTAACATTTCAAAAATCGATTGGGTTGATGAACTGAGGGACAC  900
K  V  E  N  Y  L  N  G  E  T  G  G  N  N  H  S  G  E  V  C  N  I  S  K  I  D  W  V  D  E  L  R  D  T>

TTTACATACATTGGTCGGTAACATGGCTTTACCTAAGATCGATATGATGTACTTTGATGGACAACCGGGTCAGTATTACCGTTTCATTAGTCAATTCAAT  1000
  L  H  T  L  V  G  N  M  A  L  P  K  I  D  M  M  Y  F  D  G  Q  P  G  Q  Y  Y  R  F  I  S  Q  F  N>

AGTCTTATAGAGAGTAAACTGTCAGATAAGGGCCAATTACTGTCGTATTTGTTATATTATTGCAAAGGAAAGGCCAGAACGGCAATTGAATCTTGCATTT  1100
 S  L  I  E  S  K  L  S  D  K  G  Q  L  L  S  Y  L  L  Y  Y  C  K  G  K  A  R  T  A  I  E  S  C  I>

CTTTGCCCAGCCATTCAGGCTATGATAGGGCTAAACGAATTTTATATGATTTATTTGGTAAAGAGCACCTTGTTGCGCGGGAACTAATTGCTGAGTTATT  1200
S  L  P  S  H  S  G  Y  D  R  A  K  R  I  L  Y  D  L  F  G  K  E  H  L  V  A  R  E  L  I  A  E  L  L>

AAACCATAAATCTGTCGGAAGGTCAGCTGACGTATTAACTGACTTTGCCATTAAGTTACGTAATGTATGTATAACGTTGGAGGAAATGGGATACATGTCT  1300
 N  H  K  S  V  G  R  S  A  D  V  L  T  D  F  A  I  K  L  R  N  V  C  I  T  L  E  E  M  G  Y  M  S>

GACGTTAATTCTACGGCTAATTTGGAGATAATAGTTTCATGTCTGCCACTAGAATTGCAAAATAAGTGGGCTGAAGTCGCTGATAAGATCATGATGCATG  1400
 D  V  N  S  T  A  N  L  E  I  I  V  S  C  L  P  L  E  L  Q  N  K  W  A  E  V  A  D  K  I  M  M  H>
                                                                                          D  H  D  A  W>

GGAAAGAGCCGAGCTTCGAAGAATTTGTGGTCTTGTAGAAGAGAGGGCCAGAATTGCCCGAACCCGTTATGGTAGGTTAGGTACCAGTGTAACTCAAAGG  1500
G  K  E  P  S  F  E  E  F  V  V  L  *>
 E  R  A  E  L  R  R  I  C  G  L  V  E  E  R  A  R  I  A  R  T  R  Y  G  R  L  G  T  S  V  T  Q  R>

TTCGTTAAAGGGAATTCTGAAGGCCAAGCTGATAGATCACGCTTTTCATGCAATCAAACGAGACCCCAAATGACTCAGTCAAGGGTATCAAGTTGTGCAA  1600
 F  V  K  G  N  S  E  G  Q  A  D  R  S  R  F  S  C  N  Q  T  R  P  Q  M  T  Q  S  R  V  S  S  C  A>

TTTGCTTAAGGTGATCATGAGGCGACAGATTGTCCAAGGTTGGCAAAAATGAGTGTAAGAGAAAGGAGGCAGGAGATAAGAAGACGTGGTCTATGTTACT  1700
          G  D  H  E  A  T  D  C  P  R  L  A  K  M  S  V  R  E  R  R  Q  E  I  R  R  R  G  L  C  Y>
I  C  L  R  *>

TATGTTTGAGGAAAGGTCACATAGCTATGTCATGCAACTCAGGCTTCAAGTGCGACGTCGAGAACTGCAAGGTTAGACATAATTCATTATTGCATATTGA  1800
L  C  L  R  K  G  H  I  A  M  S  C  N  S  G  F  K  C  D  V  E  N  C  K  V  R  H  N  S  L  L  H  I  D>

TGGTACTGATAACTATGTGATGAACCTAGCTAAGGATTGGAACTCCTCAAGGGTTTGTTTGGGCATCGTTCCAGTCAGACTATGCGGTCCCAAAGGATGT  1900
 G  T  D  N  Y  V  M  N  L  A  K  D  W  N  S  S  R  V  C  L  G  I  V  P  V  R  L  C  G  P  K  G  C>

TTGGAAACATATGCACTTCTAGATAGTGGTTCAGACACTTCTCTTGTATGTGAAGAATTAATTAATCAGTTGGGTATCAAAGGTAAAGAGACTTCGATAA  2000
 L  E  T  Y  A  L  L  D  S  G  S  D  T  S  L  V  C  E  E  L  I  N  Q  L  G  I  K  G  K  E  T  S  I>

GAGTGGCGACTGTGAACGGAACTACCAATTGTGAATGTTTGGAGGTAGATTTAGAGGTATTTTCATTAGATGAACGGGGTTCTATAAGGATCAACAAAGT  2100
R  V  A  T  V  N  G  T  T  N  C  E  C  L  E  V  D  L  E  V  F  S  L  D  E  R  G  S  I  R  I  N  K  V>

TTACACGACCAAGAAACTTCCGATTGATCATGCAGCACCTTTAACCGAACCCCAACTGAAAAGGTGGAAACATTTAAAGGACATAACCCTTCCGAGGTTG  2200
 Y  T  T  K  K  L  P  I  D  H  A  A  P  L  T  E  P  Q  L  K  R  W  K  H  L  K  D  I  T  L  P  R  L>

CAAAGTAATTTTGTAGGGGTATTGATTGGGTGTGATGCTCCAGATGCACATTGGGTCTTGGAACAACGTCTGGGGGACAGGAAGCATCCGTTTGCTGTGC  2300
 Q  S  N  F  V  G  V  L  I  G  C  D  A  P  D  A  H  W  V  L  E  Q  R  L  G  D  R  K  H  P  F  A  V>

GAACCCATCTCGGTTGGATGATTATAGGTCCCAAGGGGGCATCAAGGTCTCTACATCAAGTTCAGTGGTGCCATTGTTCTAATGATATTTTGCGAGATAT  2400
R  T  H  L  G  W  M  I  I  G  P  K  G  A  S  R  S  L  H  Q  V  Q  W  C  H  C  S  N  D  I  L  R  D  I>

AGAAAGATTATATAATCATGAGTTCGAGGATACAGATACGTCTCGTAATGGATATTCTGTCGAAGATAAAAGAGCTCTAGAAATAGTTAGTAATTCCTTT  2500
 E  R  L  Y  N  H  E  F  E  D  T  D  T  S  R  N  G  Y  S  V  E  D  K  R  A  L  E  I  V  S  N  S  F>

AAACTGGAAGGCGGCCATTTTCAAGTTGGTCTACCGTGGAAGTATGATAGGCCAAGTCTACCGAATAATTTGGAACTGGCTGAACGCAGACTAGAGTGTT  2600
 K  L  E  G  G  H  F  Q  V  G  L  P  W  K  Y  D  R  P  S  L  P  N  N  L  E  L  A  E  R  R  L  E  C>

TAAGGAAAAGGTTTATGAAAGATAATAGTCTTCTGCAGAAATATCAAGCTGTGATGAATAAACATTTAAGTAAGGGCTACATCATTGAAGCTAGCAAGGA  2700
L  R  K  R  F  M  K  D  N  S  L  L  Q  K  Y  Q  A  V  M  N  K  H  L  S  K  G  Y  I  I  E  A  S  K  E>

GGGATTTGACCGTGATGCTGTTTGTTGGTATATTCCTCATCATCCCGTTATCAACCCTAAAAAGCCTGGAAAACTCAGAATTGTTTTTTTGATTGTGCAG  2800
```

K A W K T Q N C F F D C A>
G F D R D A V C W Y I P H H P V I N P K K P G K L R I V F L I V Q>

CTGTCTATCAAGGATTTTCTCTTAATAATCAGTTTTTAAGAGGACCAAATACCGTCAATAGTTTATTTGGTGTACTTCTACGATTCAGATTAGGTAACAT 2900
A V Y Q G F S L N N Q F L R G P N T V N S L F G V L L R F R L G N I>
L S I K D F L L I I S F *>

AGCATTAGCCGCTGATATCGAAGAGATGTTTCTTCAAGTAAGGATACCGAGACAAGATAGGGGAGCGTTTCGTCTATTGTGGTGGGAAGATGGTGATATG 3000
A L A A D I E E M F L Q V R I P R Q D R G A F R L L W W E D G D M>

AAACGAACGGCTAAGGAATATTGTTTAACAGTTCATCCGTTTGGAGCCGTGTCCTCCCCCTTTTGCGCTAATTTCGCTCTTAAGAAGACGGTGGATATAT 3100
K R T A K E Y C L T V H P F G A V S S P F C A N F A L K K T V D I>

TCGGTAAGGAATTTAATAGAGATATCCAGGAAGTCGTAGATAATAGTTTCTATGTCGACGACTATTTAGCCTCCATTGATAATGTACAGGATGCAATTGA 3200
F G K E F N R D I Q E V V D N S F **Y V D D** Y L A S I D N V Q D A I E>

GCTGGCAAAGACCCTTGGTTTGCTTCTCAGAAAAGGTGGGTTCAGACTTACGAAGTGGATAAGTAGTTGTTTGCAAGTTCTCGAATCAATTCATCCAGAA 3300
L A K T L G L L L R K G G F R L T K W I S S C L Q V L E S I H P E>

GAGAGAGCAGAAGCCGTAGGAGAGATTGACTTTGAAAGACTTCCTACGGAACGCACGTTGGGATTATTTTGGAATACTATGGTCGATTCGTTTGACTTTA 3400
E R A E A V G E I D F E R L P T E R T L G L F W N T M V D S F D F>

AAGTCCACATACCGAAACGTCCCCTCACTAGGCGAGGTATATTATCGAGTGTAGCCTCACTTTACGACCCCTTGGGATTGTTAGCACCGTTTATACTTCC 3500
K V H I P K R P L T R R G I L S S V A S L Y D P L G L L A P F I L P>

CATGAAGCAGTTACTTCAACGTTTGGGGTAAATTGGGACTAGGATGGGACGAASAGATTCCAAATGATGAGAGCAAACGCTGGTTAGAGATTTTAAGTGA 3600
M K Q L L Q R L G *>
S S Y F N V W G K L G L G W D E X I P N D E S K R W L E I L S E>

ATTTCAGAGGGTTGAGAACGTTTGTTTTCCACGTTGTGTATTGTTTCCCGCAAATCAGATCGTTCGCTCCTGGGACTTCATATTTTCAGGKGATGCCTCG 3700
F Q R V E N V C F P R C V L F P A N Q I V R S W D F I F S G **D A S**>

GAAATGGATATGGGGGCAGTGGCATACGTCCGCTCTTTACATTTTCTGACMCTGAGGTATGTTCTCKTCTTGTTACGGCCTAAGGCAAAGATAGCCCCGT 3800
E M D M G A V A Y V R S L H F L T L R Y V L X L L R P K A K I A P>

TAAAGGYCCAATACTATMCCACYGCTTGGMACTTMCGGCAGCAGWWKTAGCAGCTCGSMWGGGATCCCAGCTGCMGKCAGAATTAGMYMTYRRGKTWYCM 3900
L K X Q Y Y X T A W X X R Q Q X *>
R X N T I P X L G T X G S X X S S S X G I P A A X R I X X X X X>

GAGSTYAAAWTTYSSACKGRYTCCATGCAYTGTCTTGMACTATATYAGRAATGRGAAAARCNCWGKKKRWWACATTCAWAKCAAWTMGSMTTKCRRCYAT 4000
R X X X X X X X H A L S X T I X X M X K X X X X T F X X X X X X X I>

YCMYMGTCTTACTAAAGTGGACCAATGGAGATTCGTACCTTCTAAAGAAAACATAGCAGACTTCGCATCCAGAGGGGTTAAGTTTAACATCGATGATGTC 4100
X X L T K V D Q W R F V P S K E N I A D F A S R G V K F N I D D V>

AAGGTATGGGAGGAGGGTCCAAGTTTTCTCAAGAAGCCGAAGGAGTGTTGGCCTGCTGTTGATATACAAGGTCCTGAACCCCACCTTTTGGAATTAAAGA 4200
K V W E E G P S F L K K P K E C W P A V D I Q G P E P H L L E L K>

AAACAATGTCCACGCATGTGATGGTTGAAGAATCCACTGTCGATCTACTTATCAATTATTATTCAGATTGGACTAGATTACTTAAGGCTGTTGCATGGTT 4300
K T M S T H V M V E E S T V D L L I N Y Y S D W T R L L K A V A W L>

GACCCGATTTAAGCTATATTTATTGATAATGCGTTCTGGTAGAACAGATCTGTCTCTACAGATGGGTATGCTCAGAGTTGATGAGTTAAATTTAGCTTGT 4400
T R F K L Y L L I M R S G R T D L S L Q M G M L R V D E L N L A C>

TTAAATCTAATTCGATATGTCCAACGCATACTATTTCGGAAAGAGATTGAAATGTTGTCGTCTGATACCATTAGCAAGGTGAAAATAACAAATTCACCGC 4500
Q I H R>
L N L I R Y V Q R I L F R K E I E M L S S D T I S K V K I T N S P>

TTACGAACTTTAAATCCAATGATGATAAACGGATTATTATGTGTCGGTGGCCGACTTCAAATTAGTTCCTGGCCCGAGTCGAGGAAGCATCCTATAATAT 4600
L R T L N P M M I N G L L C V G G R L Q I S S W P E S R K H P I I>
L T N F K S N D D K R I I M C R W P T S N *>

TACCATCGAAGCATAAAATTACAAATTTGATTCTACAGTATTATAATATTTTGGAAGGACATGTTGGGGCTACACAGGTAATGGCAACAGTCCGAGAAAA 4700
L P S K H K I T N L I L Q Y Y N I L E G **H** V G A T Q V M A T V R E K>

ATTTCGGGTGCTGAGGGGTGGTGTAGCCATGAGGAGGGTAATAAAGGATTGTGTCAGTTGTAAAAASAAGGAACGCCCGCCCCCATCCAACAACTGATGG 4800
F R V L R G G V A M R R V I K D **C** V S **C** K X K E R P P P S N N *>
R I V S V V K X R N A R P H P T T D G>

CACCACTACCTCCGAGCAGAATAAASATGGTGCCTATCCATTCTTATCASTTGGTGTTGATTATTTTGGACCCATTATGGTTAAACATGGCTASCAAGTA 4900
T T T S E Q N K X G A Y P F L S X G V **D** Y F G P I M V K H G X Q V>

CCGCAAAAGAGATATGGTTGTGTATTCACATGTTTAAGGTTGAGAGCTGTGCATCTGGAAGTTGCATATAGTTTTACCACAGACTCATTTATCATGGCAT 5000
P Q K R Y G C V F T C L R L R A V H L E V A Y S F T T D S F I M A>

TGATGAGGTTTATCATCAGGAGAGGCTATCCGAAAGAGATATACAGTGATAATGGATCAAATCTGGTGGGGGCTGAACGCGAGCTAAGAAAATGTCTTCA 5100
L M R F I I R R G Y P K E I Y S **D** N G S N L V G A E R E L R K C L Q>

GAATTGGGTGCAGGAACGTATACACTCTGATCTGCTAAGAAAAGGGATTGACCGGCATTTCAGTCCTCCGGCTGCCAGTCATTGGGGAGGAGTATGGGAG 5200
N W V Q E R I H S D L L R K G I D R H F S P P A A S H W G G V W **E**>

```
CGCATGATTCGCTCTGTACGTAGAGTATTGGGTGCTCTTGTTAAGGAACAACCTTTAACAGATGAATGCCTTGAAACTTTCATGATAGAGGCTGAGCGTA  5300
 R  M  I  R  S  V  R  R  V  L  G  A  L  V  K  E  Q  P  L  T  D  E  C  L  E  T  F  M  I  E  A  E  R>

TAATTAACAATCGACCTTTGGTCCCGGTTACAGACGACTCGAGTGATCTCGATGCAATAACACCAGCAAAACTGTTACTATTGTAGAGAGAACGTTACAG  5400
I  I  N  N  R  P  L  V  P  V  T  D  D  S  S  D  L  D  A  I  T  P  A  K  L  L  L  L  *>
                                                             H  Q  Q  N  C  Y  Y  C  R  E  N  V  T>

AACTTACTAACGTTCTATCTAATGACAGGTATTCCAAGAGATGGAAGCAAGCGAATTACTTAGCACAAGTTTTTTGGAGACGTTGGTCTAAGGAATATGT  5500
E  L  T  N  V  L  S  N  D  R  Y  S  K  R  W  K  Q  A  N  Y  L  A  Q  V  F  W  R  R  W  S  K  E  Y  V>

TTCGCTTTTGCAGCGTABATACAAATGGACCCAACTCGAGAGGAACATAAGGGAGGGTGACTTASTAATGATATGTTCTGAGTTTMTCCGAGAAAAACAA  5600
  S  L  L  Q  R  X  Y  K  W  T  Q  L  E  R  N  I  R  E  G  D  L  X  M  I  C  S  E  F  X  R  E  K  Q>

ATGGCCCCTTAGGTCTAGTACAGCAGAGTGTTACCCAAGCAAGGATGGATTAATGAGGCAGGYTAGAGCTGNAGGACAAGAAAGGGGATCCTAGHGAGAG  5700
 M  A  P  *>

ATATTAGAAAACTGTGTCTTCTTGAAGCTATAGACGGTAGGTAGCTACTCGGATCCCTAGGCGTACTGGTGTAAGTCCTAGGGATCACSAGGTTGTTAGT  5800
GTGTTGAATGCTTCTTGTTGATTGTTTGTGATGTATATACTTCGCTTCCTGCTACCTATTTACTTGTCTGTGAAAAGAACCAAGGTTCTTTTGGTCGGGA  5900
C  V  E  C  F  L  L  I  V  C  D  V  Y  T  S  L  P  A  T  Y  L  L  V  C  E  K  N  Q  G  S  F  G  R  E>

GTGTTACGGGAGAACTAAAGACATTTGGCTGATTAAAACCTTTTTACTGTACATCAAATTTACTGTGTTTTTGGATGTGTTTGTTTTACTTAACCTTTGG  6000
  C  Y  G  R  T  K  D  I  W  L  I  K  T  F  L  L  Y  I  K  F  T  V  F  L  D  V  F  V  L  L  N  L  W>

ACTTGTTTGTTTATATCATTCCTTTCGGACATTTTGGTTTCTTTTGTCATTGGTTTGGTATTTGGGAGTTTTTTGCTCCGGGAACCTACAAAATTGAAGG  6100
 T  C  L  F  I  S  F  L  S  D  I  L  V  S  F  V  I  G  L  V  F  G  S  F  L  L  R  E  P  T  K  L  K>

TTTGTTTTGTAATTGTTATAATTATTGTTGTTAGAACGACTTTGGTCGATTATTTGTGAATTGCGAATAAACTTGGTATCTTATTTGGAGTTTTGGTTCT  6200
V  C  F  V  I  V  I  I  I  V  V  R  T  T  L  V  D  Y  L  *>

CTTGCCTACTTGGGTTCGTAATTTGCGAATATCGACGGCCAGTACTTCAATAGTTGGAACGGGCAAAACTCATAATTGGACGTAACA              6300
```

Legend: Long Terminal Repeats: **boldface indigo type**
Possible promotor initiation motifs: red highlight
Direct Inverted Repeats: pink highlight
Triple Cys-His Box: underline, blue highlight
Protease active site domains: underline, pink highlight
Reverse Transcriptase seven conserved domains: underline, green highlight
RNAse H conserved DAS domain: underline, red highlight
Integrase partial zinc finger and DDE domains: underline, yellow highlight
