# Supplementary material for: The Sinbad retrotransposon from the genome of the human blood fluke, Schistosoma mansoni, and the distribution of related Pao-like elements
Source: BMC Evol Biol. 2005 Feb 23;5:20. doi: 10.1186/1471-2148-5-20 (PMC554778; doi:10.1186/1471-2148-5-20)
Supplement: Additional File 2 — "RT domain sequences of new and consensus elements used in the phylogenetic analysis". Deduced amino acid sequences of the RT domains used in the phylogenetic analysis from newly characterized elements, uncharacterized elements found within genome survey sequences, and elements for which consensus sequences were used. Accession numbers for the source sequences of each element are listed, as well as references where applicable. [file 1471-2148-5-20-S2.pdf]

**Retro-transposon**	**Accession numbers**	**RT-domain sequences**	**Key referenc**
Pao	S33901<br>AB042118<br>AB042119	LVAKGYAEPAPKTKTENRTWYLPHFAVVNPPKPEKLRVVHDAAARTRG<br>VALNDMLLKGPNLLQSLPGVIMRFRQHNITATADIKEMFIQVKLRPEDKD<br>ALRYLWRKDQRDNKPPEENRMTSLIFGASSSPSTAIYVKNLNAQKHEAT<br>HPEAAATIQNRHYVDDYLDSFKTLKDAVRITTDVRRIHEKAHFELKQWK<br>SNSLSLLETLGENENGNQVELYKPEEKTERVLGLIW	27
Boudicca	AY308018<br>AY308019<br>AY308021<br>AY308022	MDLGIIRPSNSPWASPLHMVPKKDSNDWRPTGDYRRLNAKTIPDRYPL<br>PHIHDLTATLKGTTVFSKIDLVKAYNQIPMAPDDIPKTAIITPFGLYEFLRM<br>PFGLRNAAQTFQRFIDDVFRGLNFVHAYVDDCLIASSDRESHLKHLDIVF<br>DRLQRHGITVNIQKCQIGTNSLDFLGHTI	20, 22
Sinbad	AY506538	HLSKGYIIEASKEGFDRDAVCWYIPHHPVINPXKAWKTQNCFFDCAAVY<br>QGFSLNNQFLRGPNTVNSLFGVLLRFRLGNIALAADIEEMFLQVRIPRQ<br>DRGAFRLLWWEDGDMKRTAKEYCLTVHPFGAVSSPFCANFALKKTVDI<br>FGKEFNRDIQEVVDNSFYVDDYLASIDNVQDAIELAKTLGLLLRKGGFRL<br>TKWISSCLQVLESIHPEERAEAVGEIDFERLPTERTLGLFWN	This study
D. Rerio Sinbad-like element	BX537152	IIEKGYAVRVPTEHLNRNDNKVWYIPHHGVYHPKKNKIRVVFDCTASFQ<br>GMSLNSQLLQGPNLTNTLIGVLTRFREEPIAVMADVESMFYQVKVPEED<br>TDLLRFLWWPDGNLNAPIEEYRMTVHLFGATSSPSCASYALRKTAEDR<br>KHVASQKAVDTVLNNFYVDDCLKSVSNEQEAINLVKEIQDLCLEGGFRL<br>TKWVSNNRKVLLSIPEDQRASGVKDLDLDQDSLPIERALGMQWC	This study
A. gambiae BEL-like element	XM_308737	YITLGHMTLVPREREDCSGAFYLPHHPVLKDSSSTTKVRVVFDGSAKTS<br>TGKSLNDALLVGPVVQEELLTLIIRFRKYEIALIADIEKMYRQVTMNPTDR<br>HLQRILWRFDNSQPIRTYELGTVTYGLAPSAFLATRTLIQLANDEGDKYL<br>KARSVIKENVYVDDLLAGANNIPETIELRNQLNALLQKGGFLLRKWCSN<br>APAVLADLPPELVATHSSVNFDPDESIKTLGICW	This study
C. briggsae Tas-like element	AC084491	SLEFIEEVPDESISDGPVVSYLPHHPVIKETSKSTKVRIVFDGSAKTNKVS<br>HSLNDHLHTGERLLPDIAAIMLRIRQHNILISGDIEKAFLQLVLHQTDRDA<br>TRFLWKDPTDGHLICYRYRRVPFGLKPSPYLLNKTVRTHLESYDHPWA<br>QAMINSFYVDNVFMGLDTAEEAMDFYVFAKKVFAEAQMNLCQFCSNSS<br>TVNTFFVKHEKDTTIDRNQKILGISW	This study
D. rerio Suzu-like element	BX005079	MLDRGAAVKLPESSIANWAGPVWYVSHLIAPNPHSVTTPVRLVWNSSQ<br>RFRGVSMNDLLIKGPDVLNQIRAVLLRFRSGVYAALGDIKKMYNSVWLE<br>DQEVHLHRFLWRDTENEELGEYAITRVNIGDKPAGCIAQLAMRETANLP<br>SFAHLKEERRVIQHDSYVDDILTSHNDLDQLQSIVANTELILKAGGFHLK<br>PWVFSGQSGREKSDDKSCKIKEKVMVLPNQMHDDDNKALGLGY	This study
