# Supplementary material for: The Sinbad retrotransposon from the genome of the human blood fluke, Schistosoma mansoni, and the distribution of related Pao-like elements
Source: BMC Evol Biol. 2005 Feb 23;5:20. doi: 10.1186/1471-2148-5-20 (PMC554778; doi:10.1186/1471-2148-5-20)
Supplement: Additional File 3 — "Prokaryotic genomes negative for Sinbad like elements" Table of prokaryotic genomes indicated by whole genome analysis to be devoid of Sinbad like elements. [file 1471-2148-5-20-S3.pdf]

**Microbial species/ strain**	**Accession number**	**Eubacteria or Archaea**
Aeropyrum pernix K1	NC_000854	archaea
Archaeoglobus fulgidus DSM 4304	NC_000917	archaea
Halobacterium sp. NRC-1	NC_002607	archaea
Methanocaldococcus jannaschii DSM 2661	NC_000909	archaea
Methanopyrus kandleri AV19	NC_003551	archaea
Methanosarcina acetivorans C2A	NC_003552	archaea
Methanosarcina mazei Go1	NC_003901	archaea
Methanothermobacter thermautotrophicus str. Delta H	NC_000916	archaea
Nanoarchaeum equitans Kin4-M	NC_005213	archaea
Pyrobaculum aerophilum str. IM2	NC_003364	archaea
Pyrococcus abyssi GE5	NC_000868	archaea
Pyrococcus furiosus DSM 3638	NC_003413	archaea
Pyrococcus horikoshii OT3	NC_000961	archaea
Sulfolobus solfataricus P2	NC_002754	archaea
Sulfolobus tokodaii str. 7	NC_003106	archaea
Thermoplasma acidophilum DSM 1728	NC_002578	archaea
Thermoplasma volcanium GSS1	NC_002689	archaea
Agrobacterium tumefaciens str. C58 circular	NC_003062	eubacteria
Agrobacterium tumefaciens str. C58 linear	NC_003063	eubacteria
Agrobacterium tumefaciens str. C58 circular	NC_003304	eubacteria
Agrobacterium tumefaciens str. C58 linear	NC_003305	eubacteria
Aquifex aeolicus VF5	NC_000918	eubacteria
Bacillus anthracis str. A2012	NC_003995	eubacteria
Bacillus anthracis str. Ames	NC_003997	eubacteria
Bacillus cereus ATCC 10987	NC_003909	eubacteria
Bacillus cereus ATCC 14579	NC_004722	eubacteria
Bacillus halodurans C-125	NC_002570	eubacteria
Bacillus subtilis subsp. *subtilis* str. 168	NC_000964	eubacteria
Bacteroides thetaiotaomicron VPI-5482	NC_004663	eubacteria
Bifidobacterium longum NCC2705	NC_004307	eubacteria
Bordetella bronchiseptica RB50	NC_002927	eubacteria
Bordetella parapertussis 12822	NC_002928	eubacteria
Bordetella pertussis Tohama I	NC_002929	eubacteria
Borrelia burgdorferi B31	NC_001318	eubacteria
Bradyrhizobium japonicum USDA 110	NC_004463	eubacteria
Brucella melitensis 16M I	NC_003317	eubacteria
Brucella melitensis 16M II	NC_003318	eubacteria
Brucella suis 1330 I	NC_004310	eubacteria
Brucella suis 1330 II	NC_004311	eubacteria
Buchnera aphidicola str. APS (*Acyrthosiphon pisum*)	NC_002528	eubacteria
Buchnera aphidicola str. Bp (*Baizongia pistaciae*)	NC_004545	eubacteria
Buchnera aphidicola str. Sg (*Schizaphis graminum*)	NC_004061	eubacteria
Campylobacter jejuni subsp. *jejuni* NCTC 11168	NC_002163	eubacteria

Candidatus Blochmannia floridanus	NC_005061	eubacteria
Caulobacter crescentus CB15	NC_002696	eubacteria
Chlamydia muridarum	NC_002620	eubacteria
Chlamydia trachomatis D/UW-3/CX	NC_000117	eubacteria
Chlamydophila caviae GPIC	NC_003361	eubacteria
Chlamydophila pneumoniae AR39	NC_002179	eubacteria
Chlamydophila pneumoniae CWL029	NC_000922	eubacteria
Chlamydophila pneumoniae J138	NC_002491	eubacteria
Chlamydophila pneumoniae TW-183	NC_005043	eubacteria
Chlorobium tepidum TLS	NC_002932	eubacteria
Chromobacterium violaceum ATCC 12472	NC_005085	eubacteria
Clostridium acetobutylicum ATCC 824	NC_003030	eubacteria
Clostridium perfringens str. 13	NC_003366	eubacteria
Clostridium tetani E88	NC_004557	eubacteria
Corynebacterium diphtheriae NCTC 13129	NC_002935	eubacteria
Corynebacterium efficiens YS-314	NC_004369	eubacteria
Corynebacterium glutamicum ATCC 13032	NC_003450	eubacteria
Coxiella burnetii RSA 493	NC_002971	eubacteria
Deinococcus radiodurans R1 1	NC_001263	eubacteria
Deinococcus radiodurans R1 2	NC_001264	eubacteria
Enterococcus faecalis V583	NC_004668	eubacteria
Escherichia coli CFT073	NC_004431	eubacteria
Escherichia coli K12	NC_000913	eubacteria
Escherichia coli O157:H7	NC_002695	eubacteria
Escherichia coli O157:H7 EDL933	NC_002655	eubacteria
Fusobacterium nucleatum subsp. *nucleatum* ATCC 25586	NC_003454	eubacteria
Gloeobacter violaceus PCC 7421	NC_005125	eubacteria
Haemophilus ducreyi 35000HP	NC_002940	eubacteria
Haemophilus influenzae Rd KW20	NC_000907	eubacteria
Helicobacter hepaticus ATCC 51449	NC_004917	eubacteria
Helicobacter pylori 26695	NC_000915	eubacteria
Helicobacter pylori J99	NC_000921	eubacteria
Lactobacillus johnsonii NCC 533	NC_005362	eubacteria
Lactobacillus plantarum WCFS1	NC_004567	eubacteria
Lactococcus lactis subsp. *lactis* Il1403	NC_002662	eubacteria
Leptospira interrogans serovar Lai str. 56601 I	NC_004342	eubacteria
Leptospira interrogans serovar Lai str. 56601 II	NC_004343	eubacteria
Listeria innocua Clip11262	NC_003212	eubacteria
Listeria monocytogenes EGD-e	NC_003210	eubacteria
Mesorhizobium loti MAFF303099	NC_002678	eubacteria
Mycobacterium avium subsp. *paratuberculosis* str. k10	NC_002944	eubacteria
Mycobacterium bovis AF2122/97	NC_002945	eubacteria
Mycobacterium leprae TN	NC_002677	eubacteria
Mycobacterium tuberculosis CDC1551	NC_002755	eubacteria
Mycobacterium tuberculosis H37Rv	NC_000962	eubacteria

Mycoplasma gallisepticum R	NC_004829	eubacteria
Mycoplasma genitalium G-37	NC_000908	eubacteria
Mycoplasma mobile 163K	NC_006908	eubacteria
Mycoplasma mycoides subsp. *mycoides* SC str. PG1	NC_005364	eubacteria
Mycoplasma penetrans HF-2	NC_004432	eubacteria
Mycoplasma pneumoniae M129	NC_000912	eubacteria
Mycoplasma pulmonis UAB CTIP	NC_002771	eubacteria
Neisseria meningitidis MC58	NC_003112	eubacteria
Neisseria meningitidis Z2491	NC_003116	eubacteria
Nitrosomonas europaea ATCC 19718	NC_004757	eubacteria
Nostoc sp. PCC 7120	NC_003272	eubacteria
Oceanobacillus iheyensis HTE831	NC_004193	eubacteria
Onion yellows phytoplasma OY-M	NC_005303	eubacteria
Pasteurella multocida Pm70	NC_002663	eubacteria
Photorhabdus luminescens subsp. *Laumondii* TTO1	NC_005126	eubacteria
Pirellula sp. 1	NC_005027	eubacteria
Porphyromonas gingivalis W83	NC_002950	eubacteria
Prochlorococcus marinus str. MIT 9313	NC_005071	eubacteria
Prochlorococcus marinus subsp. *marinus* str. CCMP1375	NC_005042	eubacteria
Prochlorococcus marinus subsp. *pastoris* str. CCMP1986	NC_005072	eubacteria
Pseudomonas aeruginosa PAO1	NC_002516	eubacteria
Pseudomonas putida KT2440	NC_002947	eubacteria
Pseudomonas syringae pv. tomato str. DC3000	NC_004578	eubacteria
Ralstonia solanacearum GMI1000	NC_003295	eubacteria
Rhodopseudomonas palustris CGA009	NC_005296	eubacteria
Rickettsia conorii str. Malish 7	NC_003103	eubacteria
Rickettsia prowazekii str. Madrid E	NC_000963	eubacteria
Salmonella enterica subsp. *enterica* serovar Typhi Ty2	NC_004631	eubacteria
Salmonella enterica subsp. *enterica* serovar Typhi str. CT18	NC_003198	eubacteria
Salmonella typhimurium LT2	NC_003197	eubacteria
Shewanella oneidensis MR-1	NC_004347	eubacteria
Shigella flexneri 2a str. 2457T	NC_004741	eubacteria
Shigella flexneri 2a str. 301	NC_004337	eubacteria
Sinorhizobium meliloti 1021	NC_003047	eubacteria
Staphylococcus aureus subsp. *aureus* MW2	NC_003923	eubacteria
Staphylococcus aureus subsp. *aureus* Mu50	NC_002758	eubacteria
Staphylococcus aureus subsp. *aureus* N315	NC_002745	eubacteria
Staphylococcus epidermidis ATCC 12228	NC_004461	eubacteria
Streptococcus agalactiae 2603V/R	NC_004116	eubacteria
Streptococcus agalactiae NEM316	NC_004368	eubacteria
Streptococcus mutans UA159	NC_004350	eubacteria
Streptococcus pneumoniae R6	NC_003098	eubacteria
Streptococcus pneumoniae TIGR4	NC_003028	eubacteria

Streptococcus pyogenes M1 GAS	NC_002737	eubacteria
Streptococcus pyogenes MGAS315	NC_004070	eubacteria
*Streptococcus pyogen*es MGAS8232	NC_003485	eubacteria
Streptococcus pyogenes SSI-1	NC_004606	eubacteria
Streptomyces avermitilis MA-4680	NC_003155	eubacteria
*Streptomyces coelicolo*r A3(2)	NC_003888	eubacteria
Synechococcus sp. WH 8102	NC_005070	eubacteria
Synechocystis sp. PCC 6803	NC_000911	eubacteria
Thermoanaerobacter tengcongensis	NC_003869	eubacteria
Thermosynechococcus elongatus BP-1	NC_004113	eubacteria
*Thermotoga maritim*a MSB8	NC_000853	eubacteria
Treponema denticola ATCC 35405	NC_002967	eubacteria
Treponema pallidum subsp. *pallidum* str. Nichols	NC_000919	eubacteria
*Tropheryma whipple*i TW08/27	NC_004551	eubacteria
Tropheryma whipplei str. Twist	NC_004572	eubacteria
Ureaplasma parvum serovar 3 str. ATCC 700970	NC_002162	eubacteria
Vibrio cholerae O1 biovar eltor str. N16961 I	NC_002505	eubacteria
Vibrio cholerae O1 biovar eltor str. N16961 II	NC_002506	eubacteria
Vibrio parahaemolyticus RIMD 2210633 I	NC_004603	eubacteria
Vibrio parahaemolyticus RIMD 2210633 II	NC_004605	eubacteria
Vibrio vulnificus CMCP6 I	NC_004459	eubacteria
Vibrio vulnificus CMCP6 II	NC_004460	eubacteria
Vibrio vulnificus YJ016 I	NC_005139	eubacteria
Vibrio vulnificus YJ016 II	NC_005140	eubacteria
Wigglesworthia glossinidia endosymbiont of *Glossina brevipalpis*	NC_004344	eubacteria
Wolbachia endosymbiont of *Drosophila melanogaster*	NC_002978	eubacteria
Wolinella succinogenes DSM 1740	NC_005090	eubacteria
Xanthomonas axonopodis pv. citri str. 306	NC_003919	eubacteria
Xanthomonas campestris pv. campestris str. ATCC 33913	NC_003902	eubacteria
Xylella fastidiosa 9a5c	NC_002488	eubacteria
Xylella fastidiosa Temecula1	NC_004556	eubacteria
Yersinia pestis CO92	NC_003143	eubacteria
Yersinia pestis KIM	NC_004088	eubacteria
